# Impacts of Using Peer Online Forums in Mental Health: Realist Evaluation Using Mixed Methods

**DOI:** 10.2196/79289

**Published:** 2025-10-01

**Authors:** Fiona Lobban, Neil Caton, Anna Lindroos Cermakova, Gee Collins, Zoe Glossop, Jade Haines, Steven Jones, Christopher Lodge, Karen Machin, Paul Marshall, Rachel Meacock, Tamara Rakić, Paul Rayson, Heather Robinson, Jo Rycroft-Malone, Elena Semino, Nick Shryane, Karin Tusting

**Affiliations:** 1 Spectrum Centre Department of Health Research Lancaster University Lancaster United Kingdom; 2 Berkshire Healthcare NHS Foundation Trust Berkshire United Kingdom; 3 Division of Population Health Health Services Research & Primary Care University of Manchester Manchester United Kingdom; 4 School of Computing and Communications Lancaster University Lancaster United Kingdom; 5 Faculty of Health and Medicine Lancaster University Lancaster United Kingdom; 6 Linguistics and English Language Lancaster University Lancaster United Kingdom; 7 Social Statistics University of Manchester Manchester United Kingdom

**Keywords:** mental health, online forums, impact, realist evaluation, web-based data

## Abstract

**Background:**

Peer online forums offer people experiencing mental health challenges easily accessible and anonymous support. However, little is known about the impacts of using forums, how these impacts are generated, or who might benefit from which type of forum.

**Objective:**

We aimed to develop a program theory to understand how peer online mental health forums work to help potential users, health professionals, service providers, and commissioners to decide whether to use forums and which to choose.

**Methods:**

A realist evaluation using a mixed methods, case series design in collaboration with 7 peer online mental health forums was conducted. We triangulated analysis of a large web-based survey (n=791) with in-depth realist interviews (n=52) to test and refine previously developed program theories about the impacts of using online forums. We then analyzed forum posts to identify in situ evidence for our revised theories. We only used forum posts from individuals who had freely consented to posts being shared for research. Data collection and analysis involved extensive input from our patient and public involvement group, including forum users, moderators, and senior forum staff (n=22), which met monthly for 22 two-hour-long workshops throughout the study.

**Results:**

Impacts of using peer online mental health forums were largely positive. Forums that are easy to navigate, make users feel safe to post, and are supported by well-trained moderators offering timely and sensitive responses can help people find new ways to make sense of their mental health challenges, feel understood, and accepted in the forum. This can lead to an increase in self-efficacy, a reduction in self-stigma, and increased mental well-being. Writing about experiences in a forum can itself be cathartic, but when posts have evidently been helpful to other members, posters also benefit from a sense of greater purpose and value. Negative impacts can occur if forums are difficult to navigate or if moderation is unresponsive, insensitive, or inadequate, as users can be left feeling unheard, misunderstood, or overly responsible for the welfare of others.

**Conclusions:**

Forums offer accessible and inclusive ways to effectively support mental health for many people, some of whom may have limited access to other forms of help. The impacts on users are largely positive, but care is needed to ensure forums are well designed and moderators are well trained and supported. These findings are being used to inform the co-design of a web-based moderator toolkit and design guidelines, which will be made freely available.

**Trial Registration:**

ISRCTN 62469166; https://www.isrctn.com/ISRCTN62469166

## Introduction

### Background

As demand for mental health support increases, and the availability of in-person mental health services decreases [1], peer online forums offer an accessible and anonymous source of help. Forums allow users to engage in asynchronous, primarily text-based communication, exchanging information and emotional support with those with similar experiences [2]. However, little is known about the impact of using online forums on mental health outcomes. Some positive impacts have been reported, including a reduction in depression [3], suicidal thoughts [4], and social isolation [5]. Negative impacts have also been reported, including increases in suicidal ideation [6], more negative mood [7], and worsening of body image and disordered eating patterns [8]. Given the complex, interactive nature of forums, it is likely that impacts vary depending on characteristics of users, design features of forums, and the wider context. Without a better understanding of how forums work, it is difficult for people seeking help or those signposting to decide which (if any) forum to use. It is also difficult for services wanting to host a forum to know how best to design it, or for commissioners to know which forums to invest in.

Consistent with the realist evaluation approach, which is a theory-driven approach that explores how and why interventions work or do not in specific contexts [9], we aimed to develop explanatory theories of how peer online forums “work,” for whom, and under what circumstances. Marshall et al [10] conducted a realist synthesis of literature and interview data to develop some initial program theories about how online peer mental health forums work. A program theory is an explanation of how the program under investigation (in this case, a peer online mental health forum) works. They identified 5 initial program theories, 2 of which relate to mental health outcomes: self-efficacy and social connection. This study aims to test, refine, and elaborate these 2 theories. The other 3 theory areas relate to psychological safety in forums, the role of moderation, and the impacts of forum use on other health services, and are addressed elsewhere [11,12].

Comprehensive systemic reviews show that the reported impacts of peer support interventions in general have primarily been on personal recovery outcomes (including self-efficacy and social connection), rather than clinical symptoms such as anxiety and depression (eg, Cooper et al [13]). This was also what Marshall et al [10] found for peer online forums. Therefore, we did not develop program theories about these clinical outcomes. However, guided by the Common Measures in Mental Health Science Initiative [14], we did assess levels of anxiety (using Generalized Anxiety Disorder 7 [GAD-7] [15]) and depression (using Patient Health Questionnaire 8 [PHQ-8] [16]) in forum users to understand how this population might compare to people using the large National Health Service (NHS) Talking Therapies services [17]. NHS Talking Therapies is the standard first-line offer for people seeking mental health support in England. These services offer a range of in-person sessions and web-based tools, but no online peer forum. Like forums, they are freely available and open to self-referral.

### Objectives

This study aims to (1) assess levels of distress and change over time in common clinical outcomes, including assess levels of anxiety and depression in users of online forums to understand the population, compare levels of anxiety and depression in forum users to the population seeking support from NHS Talking Therapies [17], and examine change over time for anxiety and depression outcomes in forum users; (2) test, refine, refute, and further develop initial program theories generated from a previous synthesis of the literature relevant to how recovery focused mental health impacts are generated in peer online forums.

## Methods

### Study Approach

We conducted a realist evaluation using a mixed methods, case series design in collaboration with 7 peer online mental health forums. A detailed protocol is published [18]. A realist evaluation allowed us to evaluate forums already established and in use; examine a range of impacts (positive and negative); understand how these impacts are generated; and triangulate findings across a range of methods, each of which is limited, but which together can help to build an in-depth understanding of how forums work. We triangulated data from a large sample survey designed to test relationships between key concepts in our theories; purposively sampled interviews, allowing us to explore in-depth how forums impact users; and qualitative analysis of forum posts to identify evidence of our identified mechanisms in situ. Each method has limitations and ethical challenges, but, in combination, they can enrich our theoretical understanding of how forums work. Reporting follows RAMESES II (Realist and Meta-narrative Evidence Syntheses: Evolving Standards) reporting guidelines [19].

### Role of Public and Patient Involvement Group

Our patient and public involvement (PPI) group of 22 forum users and moderators, senior forum staff, and people managing mental health challenges outside of forums met monthly for 22 two-hour-long workshops throughout the study. They were facilitated to co-design the survey and topic guides, test and refine theories, and use the emerging theories to co-design a web-based toolkit to train and support online forum moderators and forum design guidelines.

### Forums

Forums were recruited through direct contact with hosts and were eligible if they were set up by a UK-based health care provider, charity, or commercial organization with the explicit purpose of facilitating online peer-to-peer support for young people and adults experiencing mental health difficulties. To further protect the anonymity of participants, we have provided a brief description of forums in Multimedia Appendix 1, but tried to also retain the anonymity of forums by allocating each a bird name as a pseudonym, for example, “Robin” and “Magpie.”

### Initial Program Theories

Data collection was designed to test and refine the initial program theories from our realist synthesis [10] that focused on the impacts (intended and unintended) of forum use on mental health–related outcomes. These theories are broadly related to 2 key areas: self-efficacy and social connection, and are shown in Textbox 1. Consistent with realist inquiry, we have continued to draw on the context (C), mechanism (M), outcome (O) heuristic to structure our theories, which are referred to as CMO configurations. Where useful, we separated the mechanism into resource and response, as recommended by Dalkin et al [20].

**Initial program theories about the impacts of peer online forums based on previous research (these CMOs correspond to initial program theories reported in Marshall et al [10], though the numbering has changed. Corresponding CMOs here with previous publication are: CMO1=CMO1; CMO2=CMO5; CMO3=CMO18; CMO4=CMO20; CMO5=CMO21).**

**Theory area: mental health self-efficacy**

- CMO 1: In well-populated and active forums that are clearly organized (context) to allow users to find posts and receive responses that are personally relevant (mechanism—resource), users will be more likely to identify credible and actionable information that they can use to better manage their mental health (mechanism—reasoning), promoting mental health self-efficacy (outcome).
- CMO 2: When users feel safe to share their mental health experiences (context) with others whom they perceive to be nonjudgmental and as having relevant experiences (mechanism—resource), they will use the forum to reflect on their circumstances and integrate others’ perspectives into their own (mechanism—reasoning), resulting in novel and more hopeful ways of making sense of their mental health experiences (outcome).

**Theory area: social connection**

- CMO 3: When forums bring together people with similar personal experiences (context), users have access to posts that resonate with their circumstances (mechanism—resource). This normalizes their mental health experiences and validates their own reactions to similar situations (mechanism—reasoning). This can reduce self-stigma (outcome) and provide a sense of belonging (outcome).
- CMO 4: Users who share their personal experiences on the web (context) derive satisfaction (outcome) from the knowledge that their posts help others (mechanism—reasoning), particularly when others express gratitude (mechanism—resource).
- CMO 5: When forum users post messages (context) and receive timely, constructive, and empathetic responses from other users (mechanism—resource), they will feel recognized and understood (mechanism—reasoning). This will contribute to a sense of connection (outcome) with the online community (outcome), increasing forum engagement (outcome).

### Data Collection and Analysis

Data collection included a survey, interviews with forum users, and qualitative analysis of purposively sampled forum posts. Individual consent was taken for all data collected.

#### Surveys

Consistent with realist methodology guidance [21], the survey was designed to directly test our program theories, and to sample participants for interview. Two well-validated mental health outcomes were used to test anxiety (GAD-7) [15] and depression (PHQ-8 [16]), plus a bespoke item co-designed with the PPI group to assess specific contextual and mechanistic concepts within the initial program theories. A full list of survey items, including each subscale and how they were derived, is available in Multimedia Appendix 2. For multi-item concepts, Cronbach α was used to check internal reliability, and subscale means were computed (Multimedia Appendix 3).

Identical surveys were run in each forum using REDCap (Research Electronic Data Capture; Vanderbilt University) [22] at 0, 6, and 12 weeks to allow analysis to explore changes over time. Forum users aged 16 years and older were invited to take part, gave informed consent online, and received a £10 (approximately US $13.5) digital shopping voucher at each timepoint. Piloting indicated that attentively completing the survey in under 5 minutes would be impossible, so participants who did so were excluded from analysis and further follow-ups.

To compare people using forums to those seeking support from NHS Talking therapies, we used the NHS reported scoring method in which anxiety and depression (objective 1) scores for GAD-7 and PHQ-8 were summed and all participants scoring 7 or below on the GAD-7 AND 9 or below on the PHQ-8 were coded as “NotCaseness” (n=198), with the remaining coded as “caseness” (n=593) [17]. In this context, “caseness” refers to severe enough symptoms of anxiety or depression to be regarded as a clinical case needing further support. A chi-square test compared the proportion of “cases” in the survey sample to the proportion of “cases” using the NHS Talking Therapies North West region 2022-2023 database [17]. This region was chosen for convenience, as this is where the research was conducted and does not necessarily reflect where the sample was located, as all forums were national. Descriptive statistics described users’ experiences of forums and cross-sectional correlations, and 1-tailed *t* tests explored hypothesized relationships between key concepts in our program theories (objective 2). Due to the highly exploratory nature of the analyses, no corrections were made for multiple testing. Two latent growth curve models [23] were fitted to examine changes in GAD and PHQ scores over the 3 timepoints, respectively. Each model specified a latent intercept (to represent the initial state) and a latent slope (to represent linear change over time) of the 3 repeated measurements for each construct.

#### Interviews

Interview participants were recruited mainly via the survey, although some forums also advertised directly in the forum. Interviewing began after the survey was launched and continued for several months after the survey was completed. Interviews lasted between approximately 35 and 65 minutes. Interviewing stopped when there was sufficient data to refine each program theory and was consistent with the timeframe determined by the study resources. Invitations were emailed to a purposive sample of those consenting to be contacted to ensure participation across each forum and across different levels of reported forum use. Interviews were individual, semistructured, and conducted on Teams (Microsoft Corp) or by telephone by 2 research associates (ZG and PM). Participants received a £30 (approximately US $40.5) shopping voucher. Consistent with realist interviewing methodology [24], we used a combination of inductive questioning to explore participants’ experiences and deductive, theory-testing questions, asking participants to reflect on, elaborate, or refute our initial program theories. The interview topic guide (Multimedia Appendix 4) was developed in collaboration with the PPI group.

Interviews were transcribed, deidentified, and coded by the research associates (ZG and PM) into a framework built from the initial program theories using Excel (Microsoft Corp). Coded extracts were quotes detailing causal insights relevant to the key impacts specified in the initial program theories and any new impacts not previously identified. Quotes within each theory area were analyzed using retroductive reasoning (working backward from an observed phenomenon to identify the underlying mechanisms or causes that could have produced it) to further understand how these impacts were generated, and identify necessary contextual factors related to the wider social context, design of the forum, or user characteristics. Each theory was rewritten, and new theories were generated as needed. Negative impacts are understood using the same theories, demonstrating how changes in context can impact how mechanisms are activated to generate very different outcomes. The analysis was done individually, and as part of 2 full-day group workshops by core members of the analysis team (FL, ZG, HR, NC, and PM), and finally in collaboration with the whole team (including our PPI group) as part of a 1-day in-person event.

#### Forum Posts

Forum posts were used to identify evidence to support or refute our revised program theories. Ethical challenges in using online forum posts for research are explored in detail elsewhere (Lobban et al, unpublished data, 2025). Taking a very conservative position, we selected 2 forums in which users can individually choose to consent for their posts to be shared for research or not. Using only freely consented posts, we created a random sample of 120 posts from each forum that contained more than 30 words and included the phrase “thank you for...” This provided a pragmatic way to collect posts likely to describe some impacts of using the forum, though limited by a positive focus. Illustrative posts were identified and paraphrased by changes in lexis or grammatical structure, including word order, to prevent them from being traceable back to forums.

#### Triangulation

Using retroductive reasoning [25], we drew on the expertise of the PPI group and multidisciplinary research group to triangulate learning from across the 3 data streams to refine our program theories. This process began in hybrid face-to-face and web-based workshops, continued using opportunities for more reflective written feedback by email, and was finalized in our end-of-study event. In each of these, iterative versions of theories were presented alongside summarized data sources, and small groups worked to refine relevant CMO configurations.

### Ethical Considerations

Ethical approval came from the UK Health Research Authority (Solihull Research Ethics Committee) on June 20, 2022 (IRAS314029). We co-designed an ethical framework for the study published online [26]. All participants gave individual informed consent. Those taking part in the survey received a £10 (approximately US $13.5) digital shopping voucher at each timepoint, and those taking part in an interview received a £30 (approximately US $40.5) digital shopping voucher. All data were anonymized before being analyzed. No identifiable information is shared.

## Results

### Participants

A brief description of each of the 7 forums, along with levels of participation for each part of the study (survey, interview, and forum analysis), is summarized in Multimedia Appendix 1.

#### Survey

At time 1, the survey was completed by a convenience sample of 791 valid current forum users, with the most from Dunnock (n=287) and the least from Robin (n=12). At time 2, the respondent sample fell to 368, and at time 3, to 326. Although theoretically interesting to compare responses between forums (as per case series design), the highly skewed recruitment meant this was not possible, and data were analyzed as a combined sample. The sample was predominantly female (n=475, 60%), described themselves as White (n=641, 81%), and were in younger age categories, with the highest frequency in 16-24 (n=285, 36%). The majority of people came to the forum seeking help for themselves (n=546, 69%); however, a smaller but substantial number wanted help for someone else (n=237, 30%), and many also came with the intention to help others (n=229, 29%). The majority (n=427, 54%) had been using the forum for more than a month, with some (n=221, 28%) using it for a year or more. The sample was equally split between those using the forum less than or more than once a week, with a small proportion (n=712, 90%) using the forum daily. A total of 89% (n=704) had responded or liked a post in the last 6 weeks, and 64.5% (n=510) had made an original post or started a new thread. More details about survey methodology [25] and responder characteristics and forum use behavior are given in Multimedia Appendix 5.

#### Interviews

Of the 791 survey participants in the final sample, 478 consented to being contacted for an interview. A total of 214 participants were contacted, with 52 participants responding and consenting to be interviewed. Of these, 47 participants completed the interview. A further 5 forum users responded to direct adverts in the forum, totaling 52 completed interviews. Across the sample, most participants identified as White or White British (n=40, 71%), female (n=43, 77%), and were aged 16-25 years (n=22, 39%) or 26-35 years (n=14, 25%). Full demographic details by forum are available in Multimedia Appendix 6.

#### Forum Posts

No participant information is provided in relation to the 240 forum posts analyzed, as all forum data were fully anonymized before being shared with us.

### Objective 1—Levels of Distress and Impacts of Forums on Common Clinical Outcomes

Of the sample of 791 survey responders, 593 (75%) met criteria for “caseness.” Of the sample of 80,915 people completing NHS Talking Therapies in the North West UK region (2022-2023), 76,265 (94%) met criteria for “caseness.” Although levels of “caseness” in both were high, people in the survey were significantly less likely to be “cases” compared to people completing NHS Talking Therapies (*χ*^2^_1_=521.9; *P*<.001; Cramer *V*=0.08).

For the GAD-7, the latent curve model fit the data well (*χ*^2^_1_=3.0; *P*=.15). The model accounts for 70.6%, 65.8%, and 67% of the variance at t1, t2, and t3, respectively. The mean GAD7 sum score at t1 was 10.75 (SD 5.15), and the slope was –0.377 (95% CI –0.589 to –0.166) per time point, that is, a reduction of about a third of a GAD7 point on average between waves, a small net improvement. There was no significant correlation between intercept and slope (*P*=.29); that is, how high or low participants were at the start did not predict the rate of improvement.

The latent curve model of the PHQ-8 also fit the data well. (*χ*^2^_1_=1.8; *P*=.18). The model accounts for 79.5%, 66.0%, and 72.4% of the variance in PHQ-8 scores at t1, t2, and t3, respectively. The mean PHQ-8 score at t1 was 11.56 (SD 5.50). The mean slope was not significantly different from zero (slope mean –0.162, 95% CI –0.287 to 0.062), that is, there was no net improvement on average. There was no significant correlation between intercept and slope (*P*=.23); that is, how high or low participants were at the start did not predict the rate of improvement. Latent growth curves are shown in Multimedia Appendix 7.

### Objective 2—Refining Initial Program Theories

To refine our program theories, we triangulated findings from across the datasets that were relevant to each of the theory areas. The results are organized first by theory area: within each of these, illustrative findings are presented from the survey and interview, leading to a refined theory, followed by corroborating evidence from forum posts. Further detailed analyses are shown in the supplementary files.

Analysis of the survey data shows subscales means and reliability (Multimedia Appendix 3), correlations between key items and subscales in the survey (Multimedia Appendix 8), and 1-tailed *t* tests comparing respondents who never posted with those who had (Multimedia Appendix 9). Quotes from interviews are linked to each program theory in Multimedia Appendix 10. No additional forum posts are shared, as without extensive paraphrasing, they carry a greater risk of individual identification. Design recommendations based on revised program theories are shown in Textbox 1.

### Theory Area 1—Self-Efficacy

#### Survey

Consistent with CMO1, in the cross-sectional analysis at time 1, self-efficacy (selfeff_total) was statistically significantly (*P*<.001), positively associated with how helpful people found the forum (T1_help_total; *R*=0.414), and how supportive they found the moderators (T1_mod_total; *R*=0.328). Consistent with CMO2, users who felt more psychologically safe in the forum (safe_total), and were more likely to have posted at least once onto the forum (*t*_789_=2.638; *P*=.009; *d*=0.199). Posting was also associated with higher self-efficacy (*t*_419_=4.931; *P*<.001; *d*=0.407).

#### Interviews

Consistent with CMO1, there was clear evidence of people finding useful information and using this to better manage their mental health, making them feel more confident and in control. Users talked about being “taught how to take control of my mental health,” resulting in feeling “more confident and I had the ability to manage my mental health challenges.” One person commented that “I’ve a lot of skills that I’ve collected because of the forums” and that this was “collecting like a little kit to take everywhere.”

Finding personally relevant information was made easier when forum threads were clearly organized or allowed searching: “the only thing with forums is sometimes you can’t find the right stuff that you need,” and the work necessitated to overcome this may be too much for people in distress. One person who was using the forum to support a family member explained*:* “I did have a lot of things that weren’t relevant to kind of sift through so I don’t know. I think perhaps if I was looking myself and if I was dealing with anxiety that might have been a bit overwhelming.”

Forum users reported not just picking up actionable information but also being challenged to consider different perspectives on their experiences, some of which were also very useful:

It was just me coming to that realization that the way I was carrying myself, the way that I was interacting with people and the way that I was handling my mental health was not healthy at all. I wanted to emulate that ... I wanted to emulate what the community was bringing to me as a person. I wanted to emulate that open-minded attitude that they had. I wanted to emulate the optimistic attitude that they had.

Chaffinch_001

Information and new perspectives sometimes came from moderators, and sometimes from other users. Both were valued, but many users talked about the increased credibility of strategies suggested by people they perceived as having their own lived experience:

I mean with the ((Name)) workers I know that’s their job so they know what they’re talking about but I feel like they give you more professional advice whereas if it’s with someone else who’s gone through it the they might know what works better or have tried it themselves and it’s just more of an intimate sense if you get what I’m saying?

Dunnock_003

Credibility and personal applicability were further enhanced when the poster was perceived as similar to the reader, with respect to individual characteristics such as age or life circumstances:

Well, I wouldn’t want it to sounds sort of age prejudiced but I think you can relate more to people of your age type and people who are at university understand the problems they go through...

Sparrow2_003

This also applies to gender and sexuality:

I’m a nonbinary. I think I kind of appreciate the trans nonbinary people’s responses sometimes because if it’s a situation to do with how I experience dysphoria then I think sometimes that’s something that only other trans nonbinary people actually do experience in the same way.

Jay_009

In addition to reading posts, self-efficacy could be further enhanced for forum users who felt safe enough to post about their own particular experiences (CMO2). This offered more specific, actionable information directly relevant to the issue being faced.

If I’ve got an issue and I post my issue on, someone else can say, “Hey I went through that nine months ago and I did this, that and the other.” You think, “My God, why didn’t I think about that?” You might try and do those things yourself. Some things work for someone else, you’re more encouraged to try it for yourself if it’s relevant.

Magpie_100

However, to benefit from this, people need to feel safe enough to share themselves. Given the stigma associated with mental health challenges, for many users, this was difficult. Perceived lived experience in others was again a facilitator. Many users felt they would be better understood by other users in the forum who had similar lived experiences, as opposed to those bringing more professional expertise:

Yeah you do need that safety and the feeling that people understand because a lot of psychiatrists struggle really. I struggle to get them to understand where I’m coming from sometimes ... they are people that understand you. They’re not medical people going, “Oh yes you...” and they’re not trying to put labels on you because I’ve had enough labels.

Magpie_077

Although the majority view, it is important to note this attitude was not universal, with one notable exception wondering if forums might be better with “some level of actual psychiatry help through them ... Somewhere that you can place a bit of trust into what you’ve been told rather than just having Steve down the street telling you, ‘Oh, you should try magic mushrooms. It can make your depression better.’” Another contributor suggested that they would feel reassured if the moderators “had some form of training so they are prepared for the kind of content that some people might have to put on.”

This view is understandable when we delve further into the negative impacts of forums. Some people reported that reading about very relatable experiences, especially when they were traumatic, could trigger memories and even re-experiencing of their own past difficulties, causing a lot of distress:

It makes me reflect on my own and by putting myself back in that headspace I think that’s what increases the anxiety ... I think it’s like going back to what sort of kicked off I would say my social anxiety by reading other people’s experiences. It’s just not as pleasant as having a solution to feel better.

Magpie_32

This was most likely to happen when people are feeling vulnerable and when the forums were not designed with clearly labeled threads, content warnings, or premoderation, leaving users unable to manage their exposure to challenging topics:

So (Starling) has a sort of thing where you can tag it as not for ... something, but you can tag them and you know there’s a trigger in there if you don’t want to read it. Yeah like a trigger warning or something along them lines might make it a bit safer for people.

Magpie_32

Textbox 2 shows the refined theory about how peer online forums impact self-efficacy.

**Refined theory: self-efficacy.**

**Refined theory about how peer online forums impact self-efficacy**

Where forums are well-populated, active, and clearly organized, with labeled threads and content warnings (contexts), users will be more likely to find relevant and relatable posts detailing new perspectives and actionable information (mechanism—resource). When posts are from people perceived as having personal experience, and therefore high credibility, users will value and try to emulate their peers’ actions and attitudes (mechanism—reasoning). This leads to the development of strategies to better manage their mental health and more positive perspectives on their circumstances (outcome), promoting mental health self-efficacy (outcome)

When users feel safe to post (context), they receive more specific advice, relatable experiences, and encouragement, which is personalized to their particular situation (mechanism—resources). By reflecting on their own experience and integrating the perspectives of others (mechanism—reasoning), users develop novel and more hopeful ways of living (outcome).

#### Forum Posts

We analyzed forum posts to see if there was in situ evidence for the refined CMO self-efficacy. Among those expressing gratitude, we found substantial evidence that this gratuity was for receiving straightforward, actionable information, mainly in response to specific posts:

Thank you for getting back to me, have never heard of this disorder before but will look into it :).

Magpie

Gratitude was also expressed for being invited or reminded to see things from different perspectives:

Thank you for your reply. Speaking for myself only ... I have become so accustomed to down-playing myself that I don’t really see myself as a worthy person. Thank you for reminding me that I am a good person despite my mental illness.

Magpie

The fact that people ask for help within a forum is evidence that they value the lived expertise perspective, and at times, this can feel more accessible and useful than seeking help from a professional:

Thank you for reading about my problem ... Today I come here to ask for help so please if you have any solutions please HELP. Recently she felt she has depersonalization. I am not sure how I can help. She has depression and anxiety and frequently goes through panic attacks. But no doctor confirmed that, we just know. I told her to see a professional but she dismissed the idea feeling they would not take her seriously enough. Can someone help me to help her, please?

Dunnock

However, as predicted by the theory, alongside expressions of gratitude, there was also evidence that some forums may be overwhelming to navigate, particularly for people in distress:

Hello and thank you for your responses. I told my son about this site but it’s too early and he has not internet access anyway at the moment. I also think it might be too much for him, so I just tell him only some things that have been said. Wishing you all good and thanks for your help. X.

Magpie

Distress may be manageable if forums are designed in ways that clearly identify the content of threads using things like tagging, so users can decide if and when they are ready to read them:

Thank you for thinking of me. I am fine but it’s sometimes bit hard to get back to normal life. I’m learning slowly to be kind to myself. I’m really grateful for your support, it helped me through dark times but I think I might not be ready to read the thread quite yet, hope I will someday. I hope you are fine too.

Magpie

### Theory Area 2: Connection—Understanding and Acceptance

#### Overview

There were 3 initial CMO configurations under the theory area of social connection. Here, we focus on the impacts of reading (CMO3) and posting (CMO5) on stigma and connection through the mechanisms of understanding and acceptance. The third theory (CMO4) focuses on connection through helping others and is now considered separately in theory area 3 below.

#### Survey

There was good evidence that forums helped people with general loneliness and isolation. For example, 47% (n=372) felt less isolated as a result of using the forum, and 58% (n=459) felt less alone. However, this was not universal: 21% (n=166) did not feel less isolated; 18% (n=142) did not feel less alone, raising interesting questions about why these experiences differ. Survey responders reported high levels of self-stigma, with 47% (n=372) agreeing or strongly agreeing with the statement, “I feel inferior to other people because of my mental health.”

We found that people who read posts from others that resonated with their own experiences (survey item “visit_read”) described similar problems to their own (“help_same”), or conveyed understanding (help_understood); felt less isolated (“help_isolated”), and less alone (“help_alone”; neg correlation) generally, and less like an outsider (“safe_3”) and more welcome in the forum (“safe_5”) specifically. Correlations are reported in Multimedia Appendix 8.

#### Interviews

There was clear evidence that using forums could be beneficial for people in helping them to feel less alone:

I don’t feel alone anymore and that’s the best thing about it is when you go on forums and you go on these mental health support networks and you can see people, you know they’re there because they talk and they speak and it’s all words, it’s all writing you’re not alone.

Magpie_126

The benefits of reading about others’ experiences deepened when it was evident that people with similar “weird” experiences were accepted and understood, as users generalized this acceptance to themselves, even without making any posts:

It kind of made me feel less strange I suppose knowing other people are also going through it and also that they’re trying to help as well and it feels like a community kind of. [Starling_002]

Once again, the mechanisms driving connection can be activated more strongly when people post about their own experiences. It is one thing to feel accepted because you see that others share your experiences, but to post is to take a brave leap and put something very personal into the world. If responded to positively, this affords greater potential for individual acceptance, understanding, and ultimately belonging, as well as a challenge to self-stigma.

It’s helped me not to be not so ashamed. It’s helped ... this is my story, I’m a survivor. It helped me to be a warrior and not to be so ashamed and to hide it. It’s helped me to talk to people. It took me ages to say I was ill.

Magpie_077

A sense of belonging and social connection is crucial to the survival of the forum. It reinforces revisiting and generates reciprocity. This may be particularly true for people with lower levels of offline support.

I mean I’m quite blunt to be perfectly honest or people say, “I’m in a bad relationship,” and I’ll just say, “Get out.” It’s that interaction and people respond and people say, “I really like that comment. That was really helpful,” so it is definitely the interaction. Very important especially when you’re single it’s even more important. [Magpie_100]

As we saw from the survey, there is variation in how understood and accepted forum users feel, and how connected they consequently feel within the forum. We need to understand what lies behind this.

For some people, the anonymity of forums, which is crucial to creating a space safe enough to post in, may also be a barrier to feeling connected within the forum:

It’s both good and bad that no one knows each other because you can’t form any personal attachments and things because it’s not ... you don’t know anyone. For your own safety as well, you don’t know anyone so everything feels like ... it’s definitely warm. I’d describe it as welcoming but there’s not much culture on there because no one knows each other and it’s hard to directly communicate with specific people.

Dunnock_73

However, the key contextual factor across the data seemed to be how posts are responded to. People can feel rejected if their posts are not responded to quickly and sensitively. Given the asynchronous nature of forums and the limited information other forum members have available, this is a challenging task, particularly for smaller or less active forums, or forums catering to diverse populations, as at any one time may not have someone available to respond who is able to draw on similar relatable experiences.

If you’re low and you’re looking for that support if you post something and then you don’t hear something back for a day or over a day I think that would compound the feeling that you have of, “I’m low, I’m not worth anything. See, people aren’t responding to me. This is further evidence.”

Sparrow2_012

In many forums, moderators can screen or take down posts if they contravene the rules of the forum. Having a post moderated, especially if the rationale is not clear to the poster, may serve to increase rather than decrease stigma and isolation, and reduce confidence in sharing in other online and offline spaces, for example, around sexuality and transgender issues:

Talking about general mental health in a way and coping strategies yeah but being more open about my sexuality and identity not really because obviously they’ve been taken down so I don’t think I should be speaking about them in person at the time of me posting them. I mean as I got older I’ve been able to be more open about those conversations but when I probably should’ve been talking about it in the beginning and it being taken down quite quickly it kind of didn’t make you want to talk about it so it’s kind of in a way also not nice.

Dunnock_229

Textbox 3 shows the refined theory about how peer online forums foster connection through understanding and acceptance.

**Refined theory: connection through understanding and acceptance.**

**Refined** **theory about how peer online forums foster connection through understanding and acceptance**

When forums bring together people who share similar personal experiences, particularly ones stigmatized in society (context), users have access to posts that resonate for them (mechanism—resource). When they see these experiences being accepted and understood by other users and moderators, this normalizes their mental health experiences and validates their own reactions to similar situations (mechanism—reasoning), and they feel like they belong in the forum (outcome).

If users feel safe enough to make a post (context) and receive timely, constructive, and empathetic responses from other users and moderators (mechanism—resource), they will feel personally recognized, and even more understood and accepted (mechanism—reasoning), leading to a reduction in self—stigma (outcome), and a further enhanced connection with the online community (outcome), reinforcing ongoing forum engagement (outcome).

#### Forum Posts

We analyzed forum posts to see if there was in situ evidence for the refined CMO configurations driving connection through understanding and acceptance.

There was clear evidence that forums help people to feel connected. This was described as resulting from hearing the lived experience of other users, which can normalize the person’s own experiences and make them feel understood and accepted within the forum:

Thank you for sharing your thoughts and the information. I am very thankful for being in this forum. It’s very helpful to know that I am not alone and crazy. It’s been twelve months and after hearing some of the stories, I am beginning to feel I am starting to heal.

Magpie

This sense of belonging is particularly important for people who may not have someone they can easily talk to offline. It is also important in reinforcing ongoing forum engagement. When people post about their own experiences, rather than only reading about others, the nature of the replies, including sensitivity, is crucial:

Thank you for your lovely response. I feel the same, most of the things I tend to worry about don’t happen and I just create these horrible scenarios in my head of what could go wrong. I feel I do need help and change the way I think. Sometimes, I speak about this with my Mum but I am aware I can’t fully open up. She thinks I should just see my doctor. Thanks again for your supportive reply, it really helps to be here and I feel less alone x.

Dunnock

### Theory Area 3: Connection—Helping Others

#### Overview

There was substantial evidence across all 3 datasets that, in helping other people in an online forum, we also help ourselves. Helping others relies on and maintains a sense of social connection, and crucially, this active process lies at the heart of how forums are sustained. Therefore, we need to understand what facilitates this reciprocity so forums can be designed to maximize this, whilst also being aware of any potential negative impacts of helping others.

#### Survey

High levels of helping others were reported. The majority of our sample (88%) were responding to other people’s posts at least some of the time. Even on the first visit, 41% of our sample reported coming into the forum initially in order to help someone else.

#### Interviews

Overall, there was a general sense that people originally join forums seeking help, but over time they shift toward giving advice as they develop more expertise that they can then share back into the community:

I’d say a lot of them their health has improved and what they’ve done is they’ve gone on and stayed on these apps, they’ve stayed on these forums to help other people. That’s their main purpose because they’ve had probably such a bad time with their mental health and had to go through so many loopholes and they’ve had so many bad experiences or good and bad experiences they want to share that with other people because I know I do.

Magpie_126

This not only provides the relatable, actionable information needed by others to develop self-efficacy (see above) but also offers hope that positive change is possible:

It’s nice when you’re at that starting point that there are other people there that are maybe a few weeks or a few months ahead of you so you can see that all these people started in the same place and progression is possible, so that’s one of the nice things about the forums.

Dunnock_196

However, the benefits are not unidirectional. The person offering help also benefits, first through articulating their own suggestions and advice, they remind themselves to keep practicing and experimenting with these strategies, even if they had not worked every time:

My mental health definitely improved when I could help other people because I think knowing I was helping others made me realize that those techniques were actually helping and I could try using them as well whereas sometimes I’d try them once when it wasn’t working and just scrap that idea completely but I still give the idea to someone else in case it was helpful to them but then I think it made me realize I could come back to these things and try them over and over because sometimes they will work and sometimes they won’t.

Dunnock_046

Sometimes they were rewarded with other people in the forum adding their own ideas and further enhancing their repertoire of coping skills.

The main thing I get out is just having other people who may share similar experiences and supporting each other by supporting a couple of people in particular then that enhances my well-being in the fact that I feel like I’m making a difference to ... not a lot of people but just a few people and I also when I get a response back from somebody maybe give some different ideas.

Sparrow2_002

Second, many forum users reported benefitting from an increased sense of satisfaction and well-being when they shared their personal experiences, because they felt they were doing something positive and worthwhile:

I was kind of able to do a lot of work to aid and speed my recovery and being able to share that with other people it just made me feel good. It just gave me a dopamine hit I guess just from being helpful or thinking I was being helpful to others and yeah just hoping other people would feel they weren’t alone and feel that they were understood and if they found one thing helpful that I’d come across then it would be worth it.

Magpie_213

This was not just a “nice to have” feeling but provided people with a sense of self-worth that was fundamental to their own recovery. For one person, this was life-changing:

I feel like I make a difference for other people and it makes me feel less useless because I feel if I can help somebody who hasn’t got help like me then there’s still a point in me being here.

Starling_001

Both these mechanisms were amplified and reinforced when there was tangible evidence of the impact that helping was having on someone in the forum. Sometimes this was gratitude in the form of a thank you, but even more powerful was evidence of a change in someone else’s life.

I think especially when like I say you’re using a particular forum and there’s a regular user that you can keep going back to and interacting and you see them ... maybe they have an issue and you see them getting past that or you see them over time become a bit more confident.

Chaffinch_009

There was also evidence that positive impacts of helping others in an online forum could be generalized to changes in the offline world:

So I like to be able to help someone on [forum name] and having that confidence to give someone advice has really helped me to have confidence in my day to day life as well.

Dunnock_02

However, as with the previous program theories, being able to help and support people within an online forum can generate negative, as well as positive impacts, particularly for people who respond very empathically or feel a strong sense of responsibility for helping people in distress.

I think there were moments where I’d see something come through and I’d think, “I’ve got to reply to that,” and kind of dropped the things I was doing or rearranged my priorities to reply and obviously that’s not healthy but I think that’s a very ... personal issue of mine ... I was like, “I’m being useful,” so it kind of fills that need for purpose that I was seeking out so yeah it was a bit of a coping mechanism in a lot of ways but yeah better than drinking I guess.

Magpie_213

The extent to which this mechanism is activated was influenced by the perceived effectiveness of the moderation:

...if the person is in crisis ... I just feel really worried about them which is why I think it’s really good that they have the moderators then I know that person will be offered support.

Jay_009

In other instances, the relevance of the mechanism depended on the helper’s own strategies to maintain some boundaries to protect themselves:

I don’t want to be attached to people online that I don’t know basically ... I think I’d just struggle with them let’s say if they had a relapse or they were going downhill and then they didn’t reply for X amount of time on the forum, I think I’d become quite concerned and then I don’t know if that would affect me and my mental health and maybe go down.

Dunnock_196

Textbox 4 shows the refined theory about how peer online forums foster connection through helping others.

**Refined theory: connection through helping others.**

**Refined theory about how peer online forums foster connection through helping others**

When more experienced users (context) share their personal experiences and expertise on the web in response to requests from others (mechanism—resource) this increases their well-being (outcome) because it gives them a sense of purpose and value (mechanism—response) and leads them to revisit and remind themselves of how best to manage their own health (mechanism—response). When responses are met with expressions of gratitude or recipients show improvement in their lives (context), this sense of value is heightened, reinforcing further forum use and continued well-being (outcomes). However, if users read about difficult experiences that resonate for them (mechanism—resource), they may empathize deeply and want the person to receive help quickly, increasing their sense of responsibility (mechanism—response), and negatively impacting their own well-being (outcome). This responsibility is more likely to happen when the user is not confident in the moderators’ ability to respond and manage the situation (context).

#### Forum Posts

We analyzed forum posts to identify in situ evidence for the refined CMO configurations driving connection through helping others.

Unsurprisingly, the “thank you for...” posts provided a lot of evidence of people expressing gratitude to others for information, support, and acceptance they had received, but there was little evidence of the hypothesized mechanisms being overtly expressed. We can, however, infer how reinforcing it could be to be on the receiving end of some of the deep gratitude expressed, particularly for someone whose mental health may have reduced their capacity or opportunities to feel a sense of value and agency in situations in the offline world.

Thank you very much, this is so helpful! Thank you for being my friend and helping me through this. Hope all is well with you and I am so grateful you have been there through all this. I love you for your kindness and thanks for supporting me! I will act on your advice.

Dunnock

There was also evidence that, when gratitude is received, the positive impacts of this can trigger further sharing, creating a virtuous cycle.

Thank you for the appreciation, although I really didn't do a lot. Make sure you get a copy of the minutes of the meeting and don’t be afraid (if you are at the meeting) to challenge anything in the minutes you don’t agree with (safeguarding minutes are a nightmare and the minute taker doesn’t always get it all down and right ... I speak from experience!). Also make sure any actions are properly followed up. If it looks like it it being swept under the carpet (god forbid) please go to CQC for advise.

Magpie

### Theory Area 4—Catharsis and Sense-Making

This theory was not part of our initial program theories but emerged from analysis of the interview data. It describes the only mechanism specific to the online text-based context, and which does not rely on interaction between forum users. We propose that the process of writing leads to a release, externalization, and distancing from experiences that allows them to be experienced in a different way:

That’s it yeah or just to vent really sometimes. Just to put down how you’re feeling. Some people journal apparently, I don’t but it’s a way of putting it on paper and makes you feel better ... it just externalizes it from yourself. You’re sort of almost then looking in on it from other people’s point of view and you also get support which is helpful.

Dunnock_046

This processing seems particularly useful for emotive issues that are difficult to discuss in person with friends or family because they may cause distress or discomfort, or may change how the person sharing is perceived by others:

...I did talk to my friends but I don’t disclose parts of my feelings because I’m afraid of certain repercussions, so when I’m online and talking about things I consider very dark. I consider myself I have thoughts that I consider very dark. I feel more at ease knowing that it’s not going anywhere, it’s not going to damage anyone’s relationships.

Chaffinch_001

For the catharsis and sense-making to happen, people in the forum need to feel safe enough to share their very personal, sensitive reflections without censorship. This can be facilitated by anonymity within the forum.

...Eating disorders can be quite a secretive thing and that can make it isolating as well which is a problem. That’s why the forum is a good thing in a way but a lot of people don’t want to disclose that they have an eating disorder or have had an eating disorder, so having it anonymous is good in that respect.

Sparrow8_035

The key mechanism at play here does not require a response from other people in the forum, and in fact, sometimes these responses can actively inhibit this mechanism from firing.

Also I think there is an option to have comments on and comments off because sometimes people don’t want to have other people’s sympathy and empathy or they don’t want people to access what they were saying and some people do want that so I think that would be a really good option for online forums ... I think they don’t want other people to have their say in a way because that’s their experience or what they’re going through and they probably have a lot of, “Everybody’s going to be here for you, you can do this. I get what you’re doing.” Sometimes people just want to share their experience and not have other people’s experiences added on top of it. I think it’s just a preference thing for some people.

Dunnock_229

Indeed, a sense of control over what can happen in response to sharing is a key contextual facilitator:

I think because you know that nobody is going to contact you off the forum and that kind of thing. I think if you’re anonymous it is easier to share things that are a bit deeper in your mind. Does that make sense? Yeah, you’re more likely to be more vulnerable I think if you know nobody can contact you personally.

Sparrow2_84

Textbox 5 shows the new theory about how peer online forums facilitate catharsis and sense-making.

**New theory: catharsis and sense-making.**

**New theory about how peer online forums facilitate catharsis and sense-making**

When people are truly anonymous in a forum with people with shared experiences, and they can control the responses they invite from others (mechanism—resource), then they feel safe enough to post about their most challenging experiences to the forum (mechanism—response). The process of writing, and the externalization of the experiences onto the page, can bring distance and new perspectives (mechanism—response) which can fundamentally change the experience (outcome). This change can happen without any response required and may rely on being able to control responses. Creating this feeling of safety to share is particularly important for issues that are difficult to discuss face-to-face or with friends and family (context).

#### Forum Posts

We analyzed forum posts to see if there was in situ evidence for the new CMOs driving catharsis and sense-making. There was evidence of long posts, describing very personal experiences that the poster reported having struggled to share in their offline lives, and just wanted to put out into the world:

So I’m bi but I’m not out to anyone at the moment. I went to see a doctor about mental health issues and of course they asked me about my sexuality and gender. Cos I’m not out I said straight and my pronouns and it really did not feel right ugh. I want to be out cos I really want a girlfriend but I feel I can’t tell anyone. I feel like I can’t tell my Mum cause she’ll tell everyone including people I don’t want them to know cos they’re homophobic and I’m not too sure about my friendship group because they all think I’m straight. Yeah, it’s silly, I want to be out but I feel I can’t really. Anyway, thank you for reading this, even though it’s anonymous, it’s still good to be able to just say it – I’m bi!!!!

Dunnock

Some of the posts relevant to this theory started with a rationale for why the post was being written:

...I’m not expecting anyone will reply or anything, I just need a place to get these feelings out

Dunnock

Other posts ended with an explicit reflection on the purpose that writing the post had served:

...sorry, I lost the thread a bit but I sort of needed to let out what I am dealing with today.

Magpie

Less direct evidence for this mechanism can be inferred from some of these expressions of gratitude at the end of posts, which were offered to anyone who had read the post, but implied little expectation that anyone would have done so, and no request for help:

I couldn’t talk about this to anyone in real life, I am here to offload, thank you so much for reading.

Magpie

### Overall Program Theory

Although presented as 4 separate theories, these mechanisms are activated to a greater and lesser extent at different times, for different people, and in different forums. Our theories attempt to identify some of the key contextual factors influencing the most important outcomes, but we can never capture the whole experience for any individual, who may experience multiple things happening in parallel, as this interview participant describes:

If I didn’t have these forums I’d be all alone, I wouldn’t know anything. I would not have a clue what the heck was going on with my health at all. If it was not for those forums I probably wouldn’t be alive. I’d be dead. I’d have topped myself years ago. I’d have been so alone and lonely and suicidal because I was and it’s only because I’ve ... I know it sounds awful, a bit far-fetched and a bit traumatic but it’s true ... if it wasn’t for the forums and people online I’d be really ... I probably would be more depressed. These forums have helped me get over my depression.

Magpie_126

Alternatively, someone may visit the forum and experience very little happening, probably resulting in them leaving and not being in the population from which we recruited for this study.

**Figure 1:**
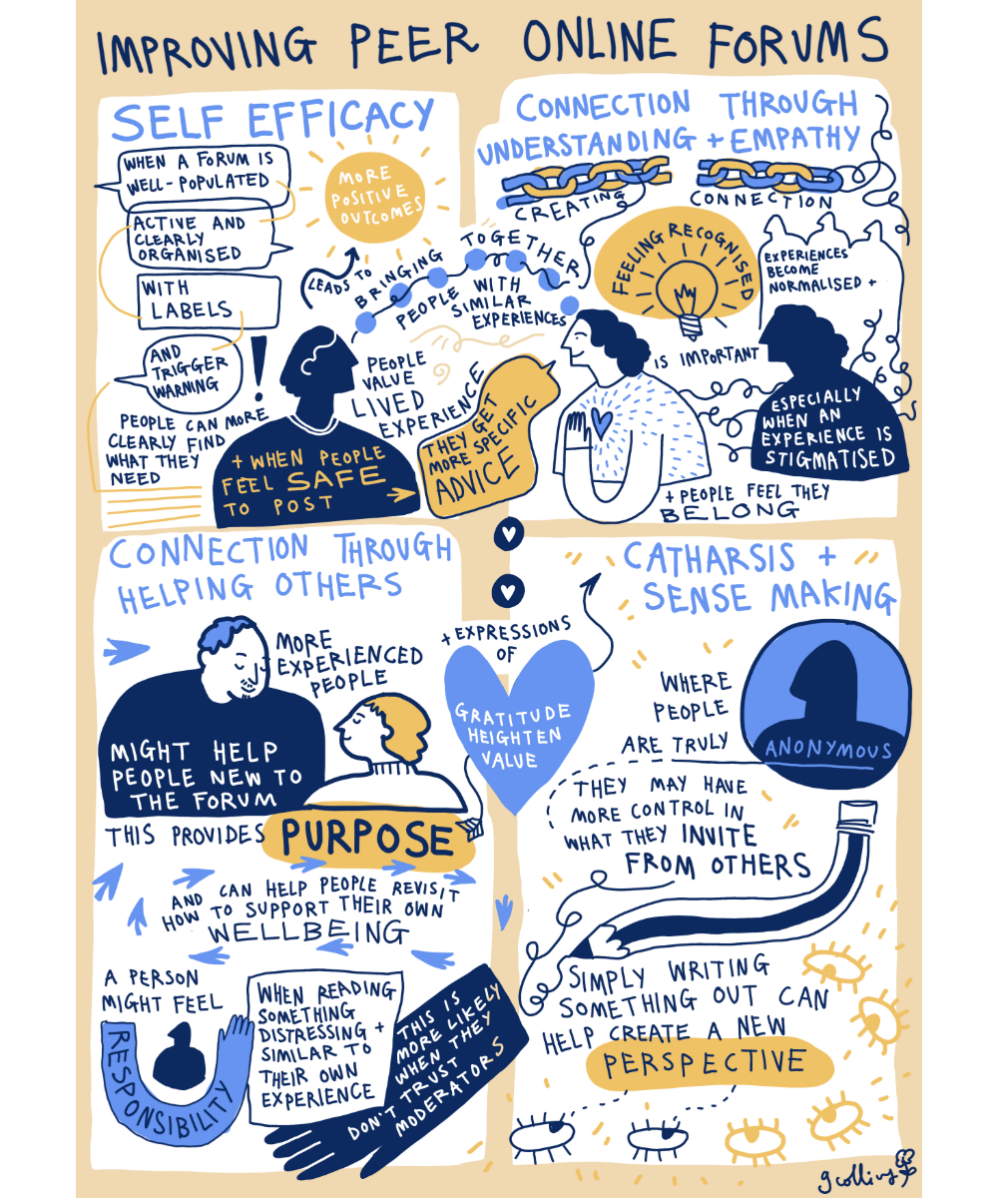
A visual representation of the overall program theory about how peer online forums work (produced by GC, who cofacilitated the patient and public involvement group).

Figure 1 is a visual representation of the overall program theory produced by GC, who cofacilitated the PPI group.

## Discussion

### Summary of Findings

A large proportion of people using online forums are experiencing clinically significant levels of anxiety or depression, though this proportion is not as high as those attending face-to-face individual therapy in the NHS. The impacts of using forums are largely positive. There was some suggestion of a small decrease in anxiety scores over time, but not for depression, though further research would be needed to test the reliability of this finding and the real-world significance of any changes. Reading posts from people with lived experience who share different perspectives and strategies for managing mental health challenges can lead to greater self-efficacy. Seeing how others are understood and accepted within the forum can be very powerful in challenging stigma and reducing isolation and loneliness. When people feel safe enough to post about their own experiences, the information, support, and acceptance they receive are more personalized, enhancing these positive impacts.

Posting brings additional benefits. The act of writing about distressing or painful experiences can be cathartic and helps externalize and create distance that affords new perspectives and insights, even without any response from others. Willingness to post also offers an opportunity to help others, as well as be helped. Helping others can serve as a reminder of one’s own wisdom and implicit understanding of mental health and generate a sense of purpose and value in being able to meaningfully contribute to the online community. Opportunities to feel valued often diminish in the offline world when people are experiencing mental health challenges, and the fact that their positive contribution comes from these challenges makes this particularly rewarding. Each of these positive impacts reinforces ongoing forum use, creating a virtuous cycle of reciprocity on which the life of the forum depends. However, each mechanism relies on design features of the forum, and to a lesser extent, the characteristics of the community of people within.

Finding new strategies and new perspectives related to specific challenges requires forums to be well populated, actively used by others with relatable experiences, and easy to navigate. People who have experienced trauma need to be able to identify posts that might be distressing to read at any moment. Well-trained, well-supported moderators are essential to balancing the need for space to allow sharing with reassurance that the well-being of forum members will be responsibly managed. Design features that promote autonomy and reinforce helping behaviors, for example, encouraging expressions of gratitude and feedback, are likely to reinforce ongoing use and reciprocity. Specific recommendations for forum design are summarized in Textbox 6.

**Forum design implications informed by revised program theories about how peer online mental health forums work (we do not propose specific rules, but list key design suggestions to consider in light of each program theory area).**

**Self-efficacy**

- Forum members are often looking for relatable experiences, so ensure forums are clearly organized into well-signposted subforums and content is easily searchable.
- Promote psychological safety through anonymity, content warnings, and transparent moderation. This will encourage people to post (as well as read), increasing the likelihood of them receiving more personalized support, and the benefit of being able to help others.
- Moderators are essential to forum support. Moderation needs to be timely and empathic. Moderators need to be well-trained and well-supported. Lived expertise and professional expertise can both be of value in moderators.

**Connection—understanding and acceptance**

- Train moderators to welcome new members to the forum and help them feel part of the community.
- Use and promote inclusive and nonstigmatizing language.
- Frame forum members as part of a community rather than site users. This can be done by encouraging and responding to feedback so that members have a sense of ownership over the way the forum works.
- Carefully consider the sensitive implementation of rules, particularly those involving hiding or removing posts. These can have unintended negative impacts if members feel misunderstood or rejected.

**Connection—helping others**

- Moderators need to find a balance between giving time to allow forum members to respond to each other, but not leaving a message without a response for too long.
- Consider ways to make it easy for forum members to express gratitude for the help they receive and share any impacts or changes they have made. Feedback can be very reinforcing to encourage ongoing reciprocation. Nontextual indicators such as emojis might support this.

**Catharsis and sense making**

- Ensure anonymity to promote safety in sharing personal details
- Introduce ways for forum members to manage the nature and timing of responses to posts, including a diary-style space that allows people to share experiences but without expecting any response.

Less prevalent in our data, but equally important to understand, are reported negative impacts. Forums that are difficult to navigate can be stressful to use and are less likely to offer helpful insights or strategies. Low levels of activity can increase the likelihood of posts being left without a response for longer and to small “cliques” forming, which are difficult for new users to infiltrate. Rather than feeling understood, accepted, and less stigmatized, users can feel ignored, rejected, and more stigmatized. Low activity reduces reinforcing feedback, creating a vicious cycle that could ultimately end the forum. Although moderators were generally highly praised, problems can arise when they are called to censor particular topics by removing or hiding posts. Balancing the needs of some users to talk about complex issues relevant to mental health, such as disordered eating or gender identity, against the possible distress this can cause to other users, is a big challenge for forum moderators. Consistent with previous work exploring forum use through the lens of social exchange theory (eg, Yan et al [27]), individual forum users are tasked with weighing up the potential benefits versus costs to them of using a forum, informed by its design and approach to moderation. These findings also build on previous research exploring how a sense of virtual community can be established through the anticipated cognitive benefits of reading posts, and social and personal integrative benefits of posting (eg, Tonteri et al [28]), providing a more in-depth understanding of how these benefits are realized and what factors can impact this.

### Interpretation of Findings

The impacts of using peer online mental health forums were largely on well-established personal recovery outcomes that are associated with enhanced quality of life and well-being. Increasing self-efficacy and social connection are associated with reduced risk of suicide (eg, Motillon-Toudic et al [29] and Chen et al [30]), and underpin NHS policies around self-management interventions [22], recovery-focused care, and social prescribing [31]. A wide literature on volunteering has shown the benefits of helping others on our own mental health and well-being [32], and the effectiveness of all psychotherapies relies to some extent on catharsis and sense making.

These impacts also have commonalities with impacts identified from other peer support approaches. Two recent umbrella reviews [13,33] suggest that individual face-to-face support from peer support workers employed in clinical services can enhance recovery-focused outcomes, and specifically, self-efficacy. A review of peer support groups for mental health [34] identified fewer studies, but similar benefits on recovery-focused outcomes. All reviews highlight the lack of high-quality evidence in peer support, and call for more research on other outcomes, including clinical ones.

This study goes beyond identifying impacts by also shedding light on the mechanisms underpinning impacts and how these are activated. Some mechanisms are likely common to all forms of peer support, including catharsis and sense making; feeling understood and accepted. Others may be more specific to online forums. Learning new strategies and perspectives, feeling understood and accepted, and reciprocity in the helping relationships within a forum are all facilitated by many people feeling safe enough to share a wide range of relatable experiences. This safety is generated in part by anonymity. In contrast, peer support workers generally work 1:1 and may not share any of their own experiences as part of the process. Face-to-face groups are not anonymous, offer a narrower range of experiences, and can be more challenging for people to attend to practically and emotionally. Thus, peer online forums offer a potentially accessible, inclusive, effective, and relatively inexpensive option for mental health support.

### Strengths and Limitations

Although realist evaluation methodology strongly advocates for triangulation across methods, it is rarely achieved in practice. The majority of studies rely on one method of data collection, and most commonly qualitative interviews [35]. This study makes a novel contribution to the development of theory about how peer online mental health forums work, and to realist methodology. Our program theories were codeveloped with input from people with lived experience, through triangulating findings across analysis of 3 large data sources (surveys, interviews, and forum posts), each with different strengths and weaknesses, collected across 7 different peer online mental health forums. Although not a representative sample, the breadth of forums increases the likelihood that the program theories are generalizable to other mental health forums, and at least in part, to other chronic health conditions. The findings have clear and actionable implications for forum design.

We examined the change in anxiety and depression over 12 weeks, but causal inference that this is due to forum use is limited. We had highly variable sample sizes across forums, limiting comparison between forums. Both the survey and interviews were limited by convenience sampling of people actively using forums, and given the high proportion who had posted (n=510, 64.5%), likely skewed toward more active members, thereby missing the views of those who have left or never used forums and who may have different views. Follow-up rates over the 12 weeks were less than half, resulting in a skewed subsample. Participants were largely White and heterosexual, which may reflect the populations using forums in the United Kingdom. Interviews were the most insightful method in this study as they allowed us to directly test initial program theories, but survey data allowed us to describe our population, gather a broad understanding of positive and negative impacts, and explore associations with cross-sectional hypothesized mechanisms. We collected a lot of forum data that provided insights into what was happening online, unfiltered through the reflexive lens of either participant or interviewer. However, the posts cannot reflect experiences of those who only ever observe in forms (generally estimated to be the majority [36]), and due to ethical concerns around the use of forum data for research, we have focused only on posts from individuals freely consenting, limiting our analysis to only 2 forums.

### Conclusions

Online peer mental health forums that are easy to navigate, make users feel safe to post, and are supported by well-trained moderators can help people find new ways to make sense of their mental health challenges, improve self-efficacy, enhance connection, and increase mental well-being. Forums offer inexpensive and inclusive ways to effectively support mental health for many people who have limited access to other forms of help. Findings are being used to inform the co-design of an online moderator toolkit and design guidelines for online forums. Further research is needed to test if these tools can enhance the positive impacts and mitigate against any negative impacts; what further adaptations can increase accessibility and use by underserved communities, such as people from ethnic minority groups; and how the emerging use of artificial intelligence might change how forums work.

## Appendix

Multimedia Appendix 1 Data collection from participating forums.

Multimedia Appendix 2 iPOF survey frequency table.

Multimedia Appendix 3 Subscale means and reliability.

Multimedia Appendix 4 Interview topic guide.

Multimedia Appendix 5 Survey demographics.

Multimedia Appendix 6 Interview participant demographics.

Multimedia Appendix 7 Latent growth curves for Generalized Anxiety Disorder 7 and Patient Health Questionnaire 8.

Multimedia Appendix 8 Correlations between items and subscales on the iPOF survey.

Multimedia Appendix 9 Comparing respondents who never posted with those who had posted using t tests.

Multimedia Appendix 10 Illustrative anonymized quotes from iPOF interviews to develop CMOs (context, mechanism, outcome) organized by theory area.

## Abbreviations

GAD-7: Generalized Anxiety Disorder 7
NHS: National Health Service
PHQ-8: Patient Health Questionnaire 8
PPI: patient and public involvement
RAMESES II: Realist And Meta-narrative Evidence Syntheses: Evolving Standards
REDCap: Research Electronic Data Capture

## Data Availability

The survey dataset generated and analyzed for this study is available in the Lancaster University Data Repository [37]. The interview and forum datasets are not publicly available due to increased risk of personal identification, but are available from the corresponding author on reasonable request.
