# Supplementary material for: Impacts of Using Peer Online Forums in Mental Health: Realist Evaluation Using Mixed Methods
Source: J Med Internet Res. 2025 Oct 1;27:e79289. doi: 10.2196/79289 (PMC12530154; doi:10.2196/79289)
Supplement: Multimedia Appendix 1 [file jmir_v27i1e79289_app1.docx]

| **Forum** | **Forum Description and access** | **Interview** | **Survey** | **N Posts in dataset (random sample from those including 30 words + containing “thank you for…”)** |
| --- | --- | --- | --- | --- |
| Starling | Discussions relating to mental health generally in the UK.  Publicly available and can be viewed without an account. Account creation requires a valid email | 3 | 213 | n/a |
| Dunnock | Designed to support the mental health of children and young people between the ages of 10 and 25.  Account required to view posts. Account creation requires a valid postcode as the forum is hosted by a private company, which is commissioned regionally. | 10 | 287 | 213125 (120) |
| Chaffinch | Aimed at connecting young people to support each other with a range of problems including mental health, and broader life challenges.  Publicly available and can be viewed without an account. Account creation requires a valid email | 3 | 49 | n/a |
| Magpie | A social networking platform that facilitates health related discussions. Non-profit organisations can create subforums.  Publicly available and can be viewed without an account. Account creation requires a valid email | 16 | 110 | 99527 (120) |
| Sparrow | NHS hosted forum for adults with specific mental health challenges, and/or seeking additional support to complement offline services.  Account required to view posts. Account creation requires a referral from the NHS service which hosts the forum. | 16 | 107 | n/a |
| Robin | A forum for anyone affected by specific mental health diagnosis, including the individual, family, and friends.  Account required to view posts. Account creation requires a valid email. | 2 | 12 | n/a |
| Jay | Online community for anyone over 16 with mental health concerns to share their experiences and engage in peer support.  Account required to view posts. Account creation requires a valid postcode or email address as the forum is hosted by a private company, which is commissioned regionally and through universities | 2 | 13 | n/a |
| Total data set |  | 52 | 791 | 220 posts (random sample) |

Note: For a full description of each forum, please visit our website <https://www.lancaster.ac.uk/health-and-medicine/research/spectrum/research/ipof/case-summaries/>
