# Supplementary material for: Impacts of Using Peer Online Forums in Mental Health: Realist Evaluation Using Mixed Methods
Source: J Med Internet Res. 2025 Oct 1;27:e79289. doi: 10.2196/79289 (PMC12530154; doi:10.2196/79289)
Supplement: Multimedia Appendix 3 [file jmir_v27i1e79289_app3.docx]

| **N** | **Scale** | **N of items** | **Scoring & Possible range*** | **Mean (SD)** | **Cronbach’s Alpha** |
| --- | --- | --- | --- | --- | --- |
| 791 | GAD-7 | 7 |  | 10.75 (5.15) | 0.887 |
|  | PHQ-8 | 8 |  | 11.56 (5.50) | 0.871 |
|  | Moderator subscale | 13 | Sum of 13x 5-point Likert scales; 0-52 | 33.70 (7.13) | 0.865 |
|  | Forum Help subscale | 9 | Sum of 9x 5-point Likert scales; 0-36 | 22.63 (5.61) | 0.853 |
|  | Safety subscale | 9 | Sum of 9x 5-point Likert scales; 0-36 | 21.59 (5.38) | 0.782 |
|  | Intensity of use | 2 | Product of 2x 4-point scales; 0-9 | 2.40 (2.08) | 0.564 |
|  | Self-efficacy | 3 | Sum of 3x 5-point Likert scales; 0-12 | 6.87 (2.48) | 0.818 |
|  | Burden | 2 | Sum of 2x 5-point Likert scales; 0-8 | 3.69 (1.92) | 0.647 |
| 759 | Mental health subscale | 8 | Sum of 8x 5-point Likert scales; 0-32 | 17.72 (4.84) | 0.75 |
| 511 | Response to posts | 5 | Sum of 5x 4-point scales; 0-15 | 9.46 (2.40) | 0.669 |

Some scales have a lower N as they only include people responding to all items, some of which are not applicable depending on previous responses *The individual items used to calculate each scale, and the mean scores for each forum, can be found in Appendix 3.
