# Supplementary material for: Impacts of Using Peer Online Forums in Mental Health: Realist Evaluation Using Mixed Methods
Source: J Med Internet Res. 2025 Oct 1;27:e79289. doi: 10.2196/79289 (PMC12530154; doi:10.2196/79289)
Supplement: Multimedia Appendix 4 [file jmir_v27i1e79289_app4.pdf]

Improving Peer Online Forums

Workstream 2 Interview Topic Guide

Action to take before the interview

Some forum users will use different terms to those used here e.g., ‘online communities’ for forums or ‘wall guides’ for moderators. Some participants may use forums with a clear focus on mental health (e.g., SHaRON) while others are more general (e.g. the Mix). Check which forum the user is recruited from prior to interview and review advertising material developed for that forum to determine the most appropriate language to use in the interview.

- Make sure the recording device is charged and ensure the location is sufficiently quiet to allow the recording device to pick up clear audio
- Introduce yourself and explain what the study is about: ‘This project is about understand people’s experiences of using online mental health forums. Our goal is to use this research to improve the effectiveness and safety of online forums.’
- Check the participant is happy to proceed based on the information in the participant information sheet and consent form
- Remind the participant of their right to confidentiality and the researcher’s obligation to end the interview and break confidentiality if risk of harm arises
- Remind the participant of their right to pause or end their participation at any time
- Introduce the idea of theory informed questioning: “as part of this interview I will ask about your experience of using forums, and for your opinions on some of the ideas and theories we have about how forums work, which have come from our work looking at previous research. This style of interview might be different to interviews you’ve been involved with before. Please feel free to share your views openly on these ideas, even if you disagree or think we’ve missed something important.”
- Offer the participant the opportunity to ask any questions about the study
- Check the participant is comfortable and prepared to conduct the interview
- Get verbal recorded consent when the recorder is turned on

Introductory questions

- Which forum do you primarily use?
- What do you tend to use the forum for?
- What motivated you to start using it?
- How frequently do you use it?

Topic 1 – Mental health self-efficacy

Exploratory questions:

- Could you describe how using the forum influences your mental health?

Theory-informed questions:

- According to some of the research we have looked at, one of the ways forums can be helpful is by giving people convenient access to information about how they can manage their mental health. What do you think of that idea?
- How do you know whether to trust the information and advice you find on mental health forums?
- Does the fact that some people share advice based on their own personal experience, influence how you view their advice?

Topic 2 – Psychological safety

Exploratory questions:

- We're interested in what makes a forum feel like a safe space. What makes a forum safe enough for you to discuss and read about issues like mental health?

Theory-informed questions:

- Do forum rules influence how safe the forum feels?
  - Are there any specific rules/examples that come to mind?
- Do moderators influence how safe the forum feels?
  - Can you think of any examples of things moderators do to promote safety?
- Some research shows that people might come across things on forums that they find upsetting. Have you ever experienced that?
  - How did you react?

  - Did that influence how much you used the forum
- One idea from previous research is that people tend to stay on forums when they feel welcomed into the online group. What is your view of that idea?
  - What influenced how welcoming the forum felt when you joined?
  - Have you ever used a forum that felt unwelcoming? What was your reaction?
- Some forums have ways to highlight that posts are about sensitive topics, for example there may be sub-forums dedicated to sensitive issues, tags on posts, or trigger warnings. Does the forum have those features?
  - Does this influence how safe the forum feels?
- Some previous research suggest that online forum conversations could encourage behaviours that could be harmful, such as self-harm or restrictive eating. Is that something you have come across online?
  - What was your reaction?
  - how is that managed by the forum/organisation?
- Some people report that they started to post in online forums because they saw other people getting friendly and helpful responses. What is your view of that?

Topic 3 – Forum access and resource use

Exploratory questions:

Have forums influenced your views on other types of mental health support?

Theory-informed questions:

Has your use of the forum led to changes in the way that you use of other types of mental health support?

Topic 4 – Forum moderation

Exploratory questions:

- We're interested in how moderators impact on forum users' experiences. What is your understanding of the role of the moderators on the forums you use?
- From your perspective, what is good forum moderation?
  - Can you think of an example where you've seen good forum moderation?
- Thinking specifically about moderators' responses to forum posts – what are the characteristics of helpful moderator responses?

    - How do you tend to react to these kinds of responses?
- And what are the characteristics of unhelpful moderator responses?
    - How do those kinds of posts impact you?

Theory-informed questions:

- I would like to get your thoughts on ideas from previous research about what a helpful moderator response might be like. How important is it that a moderator responds in the following ways:
    - Quickly
        - is it possible for moderators to respond too quickly?
    - Responds to the specific issues you have raised
    - Has a warm tone
    - Is empathetic
    - Is there anything else we have missed?

- A bad response could have the opposite characteristics. For example, a bad response might ignore or downplay your problem which is likely to make you feel disconnected from the people on the forum and maybe even less likely to post again. What is your view on that?
    - Have you seen this happen?
    - How did you/the other user react?
    -
- Has a moderator ever restricted your ability to talk about something on the forum
    - Can you think of an example?
    - How did you feel about this?
    - What impact did that have on your forum use?

Topic 5 – Social support

Exploratory questions:

- How does connecting with other people online affect your wellbeing?
- Does the forum influence your sense of being supported by other people?

Theory-informed questions:

- One thing we want to understand is what it is like for people to read posts that resonate with their experience. For example, someone may post about how they have been through the same kinds of difficulties or situations as you. How do you respond to reading those kinds of posts?
    - There's a theory that by reading posts like that, people can see that others are going through similar things, which may make people feel less isolated with their experiences, and potentially feel less stigma, too. What's your opinion on that?

- We're interested in the kinds of responses people get in online forums, and in particular, what a helpful response from other forum users looks like. When you post to the forum, what kinds of responses do you typically want from other users?
    - How important is the speed of response from other users?
    - For a response to seem helpful, some research says that it is important for users who reply to stay on topic, and directly address the original poster's concerns. What do you think?
    - A warm and empathetic tone also seems to be important for some people when they initially post to the forum. What's your view on that?
- To what extent do you feel a sense of connection with the online community on the forum?
    - Has this changed over time? If so, why?
    - Have you ever used forums where that sense of connection has been different? If so, why?
- One of the ideas we have seen in previous research is that connecting with others online may provide forum users with a feeling of belonging. Is that true in your experience?

- Do you tend to respond to other people who are looking for support on the forum?
    - How does it feel to offer that support?
    - The reason I ask is that some people find it empowering to be able to draw on their own experiences to help others. What's your view on that?

- Has using the forum influenced the way you feel about asking for support from the people around you?

- How do you decide when and how much of your own personal experiences to share when posting to the forum?

- Do you find that you are more likely to share your experiences when other people post about their own experiences? If so, why?

Topic 6 – Forum design

Exploratory questions:

- Can you describe the main features of the forum website? How do they impact your experience of the forum?

Theory-informed questions:

Two factors that seem important are anonymity and accessibility.

- How does the ability to post anonymously influence the way you use the forum?
    - Are there any other positives or negatives that you can think of?
        - Could anonymity make some people more likely to break the rules?
- Previous research suggests that one of the potential advantages of a forum is that they are usually available whenever people need them – what is your view on that?
- Does the forum you use have emojis or the ability to like posts?
    - Do you use them?
    - Why/why not?
- Does the forum have a search function?
    - Do you use it – and how?
    - (If no) would that be useful?

Debrief Instructions

- Thank the participant for taking part in the study
- Ask the participant how they found the interview and how they are feeling following their participation
- If participants feel they would benefit from further relevant information or support, direct participants to the resource list
- Provide details of how the information collected in the study will be used and give an approximate date for when the results of the study will be
- Remind participants of payment and establish a plan for this
- Provide the participant with the opportunity to ask any questions about their participation or the study in general

Before ending the meeting, engage in ordinary casual conversation at the end of the interview to help return a sense of normality
