# Supplementary material for: Impacts of Using Peer Online Forums in Mental Health: Realist Evaluation Using Mixed Methods
Source: J Med Internet Res. 2025 Oct 1;27:e79289. doi: 10.2196/79289 (PMC12530154; doi:10.2196/79289)
Supplement: Multimedia Appendix 5 [file jmir_v27i1e79289_app5.docx]

|  | Chaffinch (n=49) | | Dunnock (n=287) | | Jay (n=13) | | Magpie (n=110) | | Robin (n=12) | | Sparrow (n=107) | | Starling (n=213) | | **Total (n=791)** | |
| --- | --- | --- | --- | --- | --- | --- | --- | --- | --- | --- | --- | --- | --- | --- | --- | --- |
| **Gender** | n | % | n | % | n | % | n | % | n | % | n | % | n | % | **n** | **%** |
| Female | 23 | 46.9 | 182 | 63.4 | 9 | 69.2 | 77 | 70.0 | 10 | 83.3 | 86 | 80.4 | 88 | 41.3 | **475** | **60.05** |
| Male | 11 | 22.4 | 66 | 23.0 | 1 | 7.7 | 33 | 30.0 | 2 | 16.7 | 16 | 15.0 | 124 | 58.2 | **253** | **31.98** |
| Non-binary | 14 | 28.6 | 24 | 8.4 | 2 | 15.4 | 0 | 0.0 | 0 | 0.0 | 5 | 4.7 | 1 | 0.5 | **46** | **5.82** |
| Prefer not to say | 1 | 2.0 | 11 | 3.8 | 1 | 7.7 | 0 | 0.0 | 0 | 0.0 | 0 | 0.0 | 0 | 0.0 | **13** | **1.64** |
| Prefer to self-describe | 0 | 0.0 | 4 | 1.4 | 0 | 0.0 | 0 | 0.0 | 0 | 0.0 | 0 | 0.0 | 0 | 0.0 | **4** | **0.51** |
| **Age** | | | | | | | | | | | | | | | | |
| 16-24 | 33 | 67.3 | 208 | 72.5 | 5 | 38.5 | 0 | 0.0 | 0 | 0.0 | 19 | 17.8 | 20 | 9.4 | **285** | **36.03** |
| 25-34 | 7 | 14.3 | 51 | 17.8 | 3 | 23.1 | 7 | 6.4 | 4 | 33.3 | 25 | 23.4 | 99 | 46.5 | **196** | **24.78** |
| 35-44 | 8 | 16.3 | 16 | 5.6 | 0 | 0.0 | 5 | 4.5 | 3 | 25.0 | 30 | 28.0 | 71 | 33.3 | **133** | **16.81** |
| 45-54 | 1 | 2.0 | 5 | 1.7 | 3 | 23.1 | 23 | 20.9 | 1 | 8.3 | 13 | 12.1 | 22 | 10.3 | **68** | **8.60** |
| 55-64 | 0 | 0.0 | 4 | 1.4 | 0 | 0.0 | 45 | 40.9 | 3 | 25.0 | 12 | 11.2 | 1 | 0.5 | **65** | **8.22** |
| 65+ | 0 | 0.0 | 1 | 0.3 | 1 | 7.7 | 30 | 27.3 | 1 | 8.3 | 8 | 7.5 | 0 | 0.0 | **41** | **5.18** |
| Prefer not to say | 0 | 0.0 | 2 | 0.7 | 1 | 7.7 | 0 | 0.0 | 0 | 0.0 | 0 | 0.0 | 0 | 0.0 | **3** | **0.38** |
| **Ethnicity** | | | | | | | | | | | | | | | | |
| White | 26 | 53.1 | 237 | 82.6 | 10 | 76.9 | 98 | 89.1 | 10 | 83.3 | 95 | 88.8 | 167 | 78.4 | **643** | **81.29** |
| Black | 6 | 12.2 | 16 | 5.6 | 0 | 0.0 | 1 | 0.9 | 0 | 0.0 | 0 | 0.0 | 14 | 6.6 | **37** | **4.68** |
| Asian | 6 | 12.2 | 14 | 4.9 | 2 | 15.4 | 3 | 2.7 | 2 | 16.7 | 4 | 3.7 | 4 | 1.9 | **35** | **4.42** |
| Mixed | 5 | 10.2 | 13 | 4.5 | 0 | 0.0 | 4 | 3.6 | 0 | 0.0 | 5 | 4.7 | 28 | 13.1 | **55** | **6.95** |
| Prefer not to say. | 4 | 8.2 | 6 | 2.1 | 1 | 7.7 | 1 | 0.9 | 0 | 0.0 | 1 | 0.9 | 0 | 0.0 | **13** | **1.64** |
| Prefer to self-describe. | 2 | 4.1 | 1 | 0.3 | 0 | 0.0 | 3 | 2.7 | 0 | 0.0 | 2 | 1.9 | 0 | 0.0 | **8** | **1.01** |
| **Reason for joining** | | | | | | | | | | | | | | | | |
| I wanted to find help, advice, information or support for myself | 23 | 46.9 | 157 | 54.7 | 6 | 46.2 | 86 | 78.2 | 9 | 75.0 | 88 | 82.2 | 175 | 82.2 | **544** | **68.77** |
| I wanted to find help, advice, information or support for someone else (e.g. friends, family) | 19 | 38.8 | 107 | 37.3 | 2 | 15.4 | 6 | 5.5 | 1 | 8.3 | 5 | 4.7 | 97 | 45.5 | **237** | **29.96** |
| I wanted to offer help, advice, support, or information to other forum users | 20 | 40.8 | 80 | 27.9 | 2 | 15.4 | 12 | 10.9 | 0 | 0.0 | 20 | 18.7 | 95 | 44.6 | **229** | **28.95** |
| Other | 1 | 2.0 | 9 | 3.1 | 3 | 23.1 | 1 | 0.9 | 0 | 0.0 | 6 | 5.6 | 0 | 0.0 | **20** | **2.53** |
| **How long ago was your first visit to this forum?** | | | | | | | | | | | | | | | | |
| First visit | 5 | 11.1 | 27 | 9.7 | 1 | 8.3 | 3 | 3.4 | 0 | 0.0 | 4 | 3.9 | 26 | 12.2 | **66** | **8.34** |
| Less than one month | 22 | 48.9 | 136 | 48.9 | 5 | 41.7 | 6 | 6.7 | 4 | 40.0 | 7 | 6.9 | 74 | 34.7 | **254** | **32.11** |
| A month or more, but less than a year | 7 | 15.6 | 68 | 24.5 | 2 | 16.7 | 16 | 18.0 | 2 | 20.0 | 35 | 34.3 | 77 | 36.2 | **207** | **26.17** |
| A year or more | 11 | 24.4 | 47 | 16.9 | 4 | 33.3 | 64 | 71.9 | 4 | 40.0 | 56 | 54.9 | 36 | 16.9 | **222** | **28.07** |
| **In the last six weeks, approximately how often would you say you have visited this forum on average?** | | | | | | | | | | | | | | | | |
| First time | 6 | 12.2 | 35 | 12.2 | 1 | 7.7 | 15 | 13.6 | 0 | 0.0 | 25 | 23.4 | 17 | 8.0 | **99** | **12.52** |
| Less than one week | 21 | 42.9 | 119 | 41.5 | 6 | 46.2 | 31 | 28.2 | 0 | 0.0 | 57 | 53.3 | 82 | 38.5 | **316** | **39.95** |
| Once a week or more but less than every day | 17 | 34.7 | 116 | 40.4 | 5 | 38.5 | 50 | 45.5 | 7 | 58.3 | 19 | 17.8 | 92 | 43.2 | **306** | **38.69** |
| Every day | 5 | 10.2 | 17 | 5.9 | 1 | 7.7 | 14 | 12.7 | 5 | 41.7 | 6 | 5.6 | 22 | 10.3 | **70** | **8.85** |
| **In the last six weeks, approximately how much time have you usually spent on this forum on each visit?** | | | | | | | | | | | | | | | | |
| Less than 5 minutes | 8 | 16.3 | 17 | 5.9 | 2 | 15.4 | 18 | 16.4 | 0 | 0.0 | 44 | 41.1 | 8 | 3.8 | **97** | **12.26** |
| More than 5 minutes but less than half an hour | 23 | 46.9 | 105 | 36.6 | 7 | 53.8 | 61 | 55.5 | 4 | 33.3 | 42 | 39.3 | 63 | 29.6 | **305** | **38.56** |
| More than half an hour, but less than hour | 11 | 22.4 | 140 | 48.8 | 4 | 30.8 | 24 | 21.8 | 2 | 16.7 | 18 | 16.8 | 105 | 49.3 | **304** | **38.43** |
| An hour or more | 7 | 14.3 | 25 | 8.7 | 0 | 0.0 | 7 | 6.4 | 6 | 50.0 | 3 | 2.8 | 37 | 17.4 | **85** | **10.75** |
