# Supplementary material for: Impacts of Using Peer Online Forums in Mental Health: Realist Evaluation Using Mixed Methods
Source: J Med Internet Res. 2025 Oct 1;27:e79289. doi: 10.2196/79289 (PMC12530154; doi:10.2196/79289)
Supplement: Multimedia Appendix 6 [file jmir_v27i1e79289_app6.docx]

|  | Count (total = 52) | % |
| --- | --- | --- |
| **Gender** |  |  |
| Female | 39 | 75% |
| Male | 9 | 17% |
| Non-binary | 1 | 2% |
| Preferred not to say | 2 | 4% |
| Self-defined (transgender male) | 1 | 2% |
| **Age group** |  |  |
| 16 - 24 | 11 | 21% |
| 25 - 34 | 12 | 23% |
| 35 - 44 | 7 | 13% |
| 45 - 54 | 6 | 12% |
| 55 - 64 | 10 | 19% |
| 65+ | 6 | 12% |
| **Ethnicity** |  |  |
| White/White British | 43 | 82% |
| Black/African/Caribbean/Black British | 2 | 4% |
| Asian/Asian British | 3 | 6% |
| Mixed/Multiple ethnic groups | 1 | 2% |
| Other ethnic group | 1 | 2% |
| Preferred not to say | 2 | 4% |
