# Supplementary figures and images for: Impacts of Using Peer Online Forums in Mental Health: Realist Evaluation Using Mixed Methods
Source: J Med Internet Res. 2025 Oct 1;27:e79289. doi: 10.2196/79289 (PMC12530154; doi:10.2196/79289)

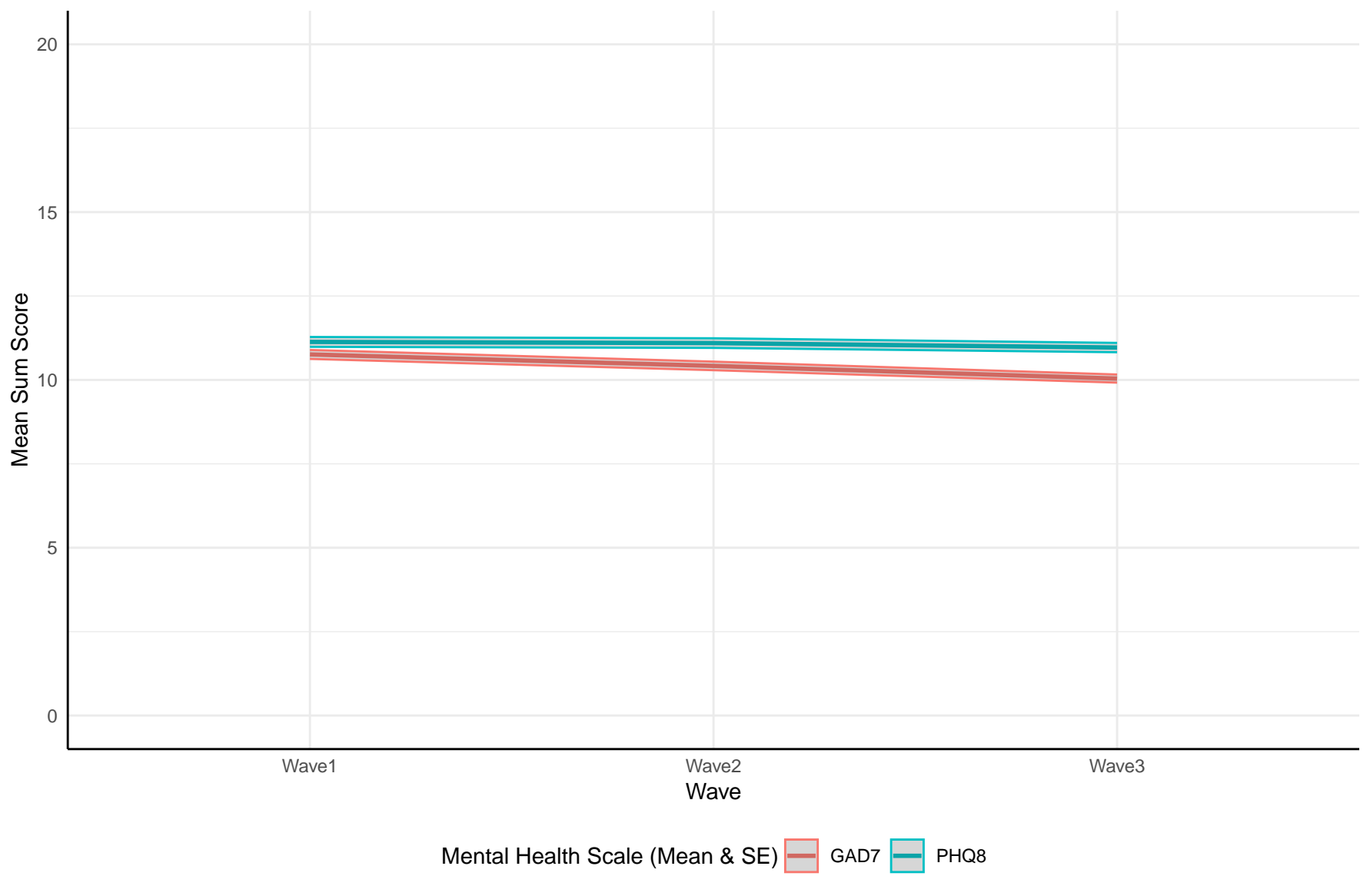

Supplement: Multimedia Appendix 7 [file jmir_v27i1e79289_app7.pdf]
