# Supplementary material for: Impacts of Using Peer Online Forums in Mental Health: Realist Evaluation Using Mixed Methods
Source: J Med Internet Res. 2025 Oct 1;27:e79289. doi: 10.2196/79289 (PMC12530154; doi:10.2196/79289)
Supplement: Multimedia Appendix 8 [file jmir_v27i1e79289_app8.docx]

|  |  | selfeff_total | help_total | mh_inferior | burden_total | Help_isolated | Help_alone | Safe_3 | Safe_5 | Visit_again |
| --- | --- | --- | --- | --- | --- | --- | --- | --- | --- | --- |
| T1_mod_total | *R* | .328^**^ |  |  | -.152^**^ | .275^**^ | -.444^**^ |  |  |  |
|  | Sig. | <.001 |  |  | <.001 | <.001 | <.001 |  |  |  |
|  | N | 791 |  |  | 791 | 791 | 791 |  |  |  |
| T1_help_total | *R* | .414^**^ |  | -.060 |  |  |  |  |  |  |
|  | Sig. | <.001 |  | .091 |  |  |  |  |  |  |
|  | N | 791 |  | 791 |  |  |  |  |  |  |
| t1_intensity | *R* |  | .284^**^ |  |  |  |  |  |  |  |
|  | Sig. |  | <.001 |  |  |  |  |  |  |  |
|  | N |  | 791 |  |  |  |  |  |  |  |
| t1_visit_post | *R* |  | .183^**^ |  |  |  |  |  |  |  |
|  | Sig. |  | <.001 |  |  |  |  |  |  |  |
|  | N |  | 791 |  |  |  |  |  |  |  |
| t1_visit_receive | *R* |  | .121^**^ |  |  |  |  |  |  |  |
|  | Sig. |  | .005 |  |  |  |  |  |  |  |
|  | N |  | 526 |  |  |  |  |  |  |  |
| t1_posts_response | *R* |  | .559^**^ |  | -.076 | .269^**^ | -.433^**^ | -.424^**^ | .499^**^ | .352^**^ |
|  | Sig. |  | <.001 |  | .086 | <.001 | <.001 | <.001 | <.001 | <.001 |
|  | N |  | 511 |  | 511 | 511 | 511 | 511 | 511 | 511 |
| T1_help_same | *R* |  |  | -.025 |  | .370^**^ | -.343^**^ | -.285^**^ | .341^**^ |  |
|  | Sig. |  |  | .479 |  | <.001 | <.001 | <.001 | <.001 |  |
|  | N |  |  | 791 |  | 791 | 791 | 791 | 791 |  |
| T1_help_understood | *R* |  |  | -.015 |  | .397^**^ | -.296^**^ | -.301^**^ | .408^**^ |  |
|  | Sig. |  |  | .677 |  | <.001 | <.001 | <.001 | <.001 |  |
|  | N |  |  | 791 |  | 791 | 791 | 791 | 791 |  |
| T1_visit_read | *R* |  |  | .023 |  | .397^**^ | -.202^**^ | -.203^**^ | .195^**^ |  |
|  | Sig. |  |  | .512 |  | <.001 | <.001 | <.001 | <.001 |  |
|  | N |  |  | 791 |  | 791 | 791 | 791 | 791 |  |
| Safe_total | *R* |  |  |  | -.206^**^ | .270^**^ | -.487^**^ |  |  |  |
|  | Sig. |  |  |  | <.001 | <.001 | <.001 |  |  |  |
|  | N |  |  |  | 791 | 791 | 791 |  |  |  |

** indicates significance at the *p*<.001 level.
