# Supplementary material for: Impacts of Using Peer Online Forums in Mental Health: Realist Evaluation Using Mixed Methods
Source: J Med Internet Res. 2025 Oct 1;27:e79289. doi: 10.2196/79289 (PMC12530154; doi:10.2196/79289)
Supplement: Multimedia Appendix 9 [file jmir_v27i1e79289_app9.docx]

| Measure | Group | Mean (SD) | T-test | Cohen’s d |
| --- | --- | --- | --- | --- |
| Safety scale | Never posted (n=265) | 20.88 (5.63) | *t*(789)=2.638, *p*=.009 | 0.199 |
|  | Posted at least a few times in past 6 weeks (n=526) | 21.95 (5.22) |  |  |
| Self-efficacy subscale | Never posted (n=265) | 6.21 (2.89) | *T*(419)=4.931, *p*<.001 | 0.407 |
|  | Posted at least a few times in past 6 weeks (n=526) | 7.21 (2.18) |  |  |
