# Supplementary material for: Impacts of Using Peer Online Forums in Mental Health: Realist Evaluation Using Mixed Methods
Source: J Med Internet Res. 2025 Oct 1;27:e79289. doi: 10.2196/79289 (PMC12530154; doi:10.2196/79289)
Supplement: Multimedia Appendix 10 [file jmir_v27i1e79289_app10.docx]

| Theory Area | Original theory | Illustrative quotes | Revised theory |
| --- | --- | --- | --- |
| Theory area 1: Self-efficacy | CMO 1: in well-populated and active forums that are clearly organized (context) to allow users to find posts and receive responses that are personally relevant (mechanism—resource), users will be more likely to identify credible and actionable information that they can use to better manage their mental health (mechanism—reasoning), promoting mental health self-efficacy (outcome). | I think you know t*hey* ***taught me how to take control of my mental health*** and they provided me with some resources, support and I felt ***more confident and I had the ability to manage my mental health challenges*** so that’s why I felt empowered.  WS2_PM_Us_Jay_001 (ZG)     There’s lots of people who’ve got these problems and like I say it’s something – and you might read on there, which [I might have: 0:14:34.6] some of them might be going through something, ***somebody suggests something and you can relate that to your own life*** and you can kind of in a roundabout way with yourself sort of thing.  WS2_PM_Us_Sparrow2_023 (ZG)    Also, I think some of the information that I found was helpful for me and the way that people – ***I’ve got a lot of skills that I’ve collected because of the forums*** like different ways to do mindfulness because I’m not a person who likes to do breathing for mindfulness so finding things that were more active for mindfulness that was quite helpful and that’s the thing that I got from the forum but also about ***collecting like a little kit to take everywhere*** with me in case I get overwhelmed or anything  WS2_PM_Us_Dunnock_229 (ZG)    ***the only thing with forums is sometimes you can’t find the right stuff that you need*** or the help but I’ve also talked to peers one to one and everything and it’s helped me to get support and everything**.** WS2_PM_Us_Magpie_077 (ZG)    so I was trying to find – as I said in the beginning, trying to find some kind of stories that would have parallels to my sister’s that I might be able to share with her or find some information that I could then rephrase to her that might be useful but I did find it quite difficult because it was just in those broad categories of anxiety. I think it was anxiety and OCD. I don’t think anxiety was even a standalone category. ***I did have a lot of things that weren’t relevant to kind of sift through so I don’t know. I think perhaps if I was looking myself and if I was dealing with anxiety that might have been a bit overwhelming***,   WS2_PM_Us_Chaffinch_022 (ZG)    It just made me realise you know what the way that I carried myself it was not healthy at all. They made me realise you know what – obviously they didn’t outright say it. It was just me coming to that realisation that the way I was carrying myself, the way that I was interacting with people and the way that I was handling my mental health was not healthy at all. I wanted to emulate that – I wanted to emulate what the community was bringing to me as a person. I wanted to emulate that open-minded attitude that they had. ***I wanted to emulate the optimistic attitude that they had***. I’ve also wanted to emulate the problem – the way that they approached those problems as well and I just wanted to have that positive outlook and see how I can help me as a person, how I can emulate that into my life, so I can be a better person than I was back then, how I could continue to grow, so I feel like they helped me to grow to become a better person than I was before and they still help me grow from that point forward.  WS2_PM_Us_Chaffinch_001 (ZG)    Just because – I mean with the ((Name)) workers [I know: 0:11:35.4] that’s their job so they know what they’re talking about but I feel like they give you more professional advice whereas ***if it’s with someone else who’s gone through it the they might know what works better or have tried it themselves*** and it’s just more of an intimate sense if you get what I’m saying?   WS2_PM_Us_Dunnock_003  because I have autism so for me I think sometimes asking a – like a neurotypical response might not be because I’m a nonbinary I think I kind of appreciate the trans nonbinary people’s responses sometimes ***because if it’s a situation to do with how I experience dysphoria then I think sometimes that’s something that only other trans nonbinary people actually do experience in the same way***.   WS2_ZG_Us_Jay_009 (ZG)    Well, I wouldn’t want it to sounds sort of age prejudiced but I think ***you can relate more to people of your age*** type and people who are at university understand the problems they go through with exams and perhaps bullying whereas older people – my generation we didn’t have that when I was younger so it’s a bit more difficult to relate to it  WS2_PM_Us_Sparrow2_003 (ZG)    The thing ***that’s a shame really*** about the [S] forum in particular and I’ve talked at length to ((Name[person involved in setting [Starling] up about this because she sort of says it’s not what it was intended for but ***the vast majority of people who post are psychology assistants and there is very little kind of peer commentary***.  WS2_PM_Us_Sparrow8_019 (ZG)    So perhaps ***strangely the service users that have responded occasionally... it’s actually been far more useful*** in that there’s not the tea and sympathy that goes with it because it can actually – ***particularly when it’s coming from somebody who is a staff member within the service it can actually sound quite trite***. There’s a sort of lack of sincerity that almost, ‘Okay this is the right thing that I ought to be saying to this person who’s coming across as in a state of distress.’ Whereas I think with fellow service users it tends to be a kind of like, ‘Yeah, we know it’s crap but this is actually the best way to handle it. This is what I found helpful, you might want to give it a go**.’** WS2_PM_Us_Sparrow8_019 (ZG) | **CMO – Self efficacy**  Where forums are well-populated, active, and clearly organised, with labelled threads and content warnings (contexts), users will be more likely to find relevant and relatable posts detailing new perspectives and actionable information, (mechanism resource). When posts are from people perceived as having personal experience, and therefore high credibility, users will value and try to emulate their peers’ actions and attitudes (mechanism reasoning). This leads to the development of strategies to better manage their mental health and more positive perspectives on their circumstances (outcome), promoting mental health self-efficacy (outcome).  When users feel safe to post (context), they receive more specific advice, relatable experiences, and encouragement, which is personalised to their particular situation (mechanism resources). By reflecting on their own experience and integrating the perspectives of others (mechanism – reasoning), users develop novel and more hopeful ways of living (outcome). |
|  | CMO 2: when users feel safe to share their mental health experiences (context) with others whom they perceive to be nonjudgmental and as having relevant experiences (mechanism—resource), they will use the forum to reflect on their circumstances and integrate others’ perspectives into their own (mechanism—reasoning), resulting in novel and more hopeful ways of making sense of their mental health experiences (outcome). | I mean last week there was one on there, some lady was having a procedure done and she suffers with claustrophobia and I just said, ‘I’m a yoga teacher’ – because I’ve had a couple of MRI scans over the last few years, ‘I find those really claustrophobic so I just use this breathing technique,’ and so she took on board and said, ‘Oh that’s really kind, thank you very much. I will do that,’ and then we went off on a tangent and talked about this Buddhist coach which was really nice. Then another person came on and joined in and just said, ‘Have you thought about trying a blindfold?’ ***which was just the most amazing piece of advice that I’d never ever thought of*** so that on a real positive level. WS2_PM_Us_Magpie_073 (ZG)    Probably ***that I did actually leave the relationship so yeah it prompted me to do that*** because I was sort off – not in the relationship still but I was kind of like, ‘Oh we’ll still live together,’ and that sort of thing and actually it did make me realise that wasn’t going to be a possibility. WS2_PM_Us_Goldfinch_001    It’s ***definitely helpful because it makes me realise that there are people that can actually get through it, and it makes me realise that I don’t have to figure it out by myself because I can speak to people who do know what they’re talking abou***t WS2_PM_Us_Dunnock_046 (PM)    Yeah you do need that safety and the feeling that people understand because a lot of psychiatrists struggle really. I struggle to get them to understand where I’m coming from sometimes... ***they are people that understand you.*** They’re not medical people going, ‘Oh yes you…’ and ***they’re not trying to put labels on you because I’ve had enough labels***. WS2_PM_Us_Magpie_077 (ZG)    if I’ve got an issue and I post my issue on, someone else can say, ‘Hey I went through that nine months ago and I did this, that and the other.’ ***You think, ‘My God, why didn’t I think about that?’ You might try and do those things yourself.*** Some things work for someone else, you’re more encouraged to try it for yourself if it’s relevant. WS2_PM_Us_Magpie_100 (ZG)    I: Say if you’re posting a message or you’re starting a post asking for advice would you rather get responses from other users or staff?   R: I think ***users because then I know they’ve gone through it [were sort of better: 0:16:36.3] and kind of know what to expect***. WS2_PM_Us_Magpie_178 (ZG)    Because I think if you are dealing with people who are struggling through mental illness and that, I don’t think it’s necessarily about showing emotion, i***t’s about being rational where people can’t necessarily be rationa***l. You can say, ‘Oh I’m really sorry that you are feeling unhappy. Could you do this? Could you do that?’ I think it’s got to be quite solution based....***I get the emotional stuff and the validation from the users but don’t necessarily need that from the moderators*** WS2_PM_Us_Sparrow2_064 (ZG)    the ideal forum would be. Probably some level of ***actual psychiatry help*** through them, maybe a moderator or two that actually can help a bit more, point you in the right direction of things. Somewhere that ***you can place a bit of trust into what you’ve been told rather than just having Steve down the street telling you***, ‘Oh, you should try magic mushrooms. It can make your depression better.’  WS2_PM_Us_Magpie_032 (ZG) |  |
|  | Negative impacts | I’m a really empathetic person so when I read somebody else’s situation ***I really put myself in their shoes and it can actually make how I’m feeling worse***. I feel like I reflect more on my own anxiety and I sort of increase my anxiety because of that WS2_PM_Us_Magpie_032 (ZG)  One time the main thing I was trying to get some advice on was social anxiety and that type of thing and I think was reading comments surrounding social anxiety and things that people have come across and that’s happened to them, that’s got them feeling anxious about social aspects basically. It makes me reflect on my own and by ***putting myself back in that headspace I think that’s what increases the anxiety***... I think it’s like going back to what sort of kicked off I would say my social anxiety by reading other people’s experiences. It’s just not as pleasant as having a solution to feel better. WS2_PM_Us_Magpie_032 (ZG)  so (Starling) has a sort of thing where you can tag it as not for - something but ***you can tag them and you know there’s a trigger*** ***in there*** if you don’t want to read it. Yeah like a trigger warning or something along them lines might ***make it a bit safer for people*** who – like for example how I’ve got the thing with my anxiety reading somebody’s thing of anxiety. ***If somebody’s got a thing with suicide, you don’t really want to be reading about suicide if it can make you feel more suicidal*** do you know what I mean so it’s definitely good to have a sort of tag in there for it. WS2_PM_Us_Magpie_032 (ZG)  all practitioners actually will ***put a little trigger warning*** right at the top to explain what’s going to be in that forum before anyone has to read it because even if it’s not something that ***might particularly be triggering or something you’re not supposed to say for example, they still would add it anyway because different things trigger different people. Whereas lots of places don’t actually put that unless it’s going to be something that’s massively triggering so you don’t realise until you’re read it***. WS2_PM_Us_Dunnock_046 (PM) |  |
| Theory area 2: social connection | CMO 3: when forums bring together people with similar personal experiences (context), users have access to posts that resonate with their circumstances (mechanism—resource). This normalizes their mental health experiences and validates their own reactions to similar situations (mechanism—reasoning). This can reduce self-stigma (outcome) and provide a sense of belonging (outcome). | and then I found this forum and I started looking and asking questions and everything and it actually helped me to understand. ***I didn’t feel so alone and it just started me journey of starting to understand what I’d been though and everything***. I’ve also been able to help other mothers who go through it but some of the stuff –WS2_PM_Us_Magpie_077 (ZG)  Somebody that’s never suffered from it I don’t think would be so understanding, would realise the implications of what it can do to people...***I think they’ve got empathy***. I think they have quite a deep understanding of how it makes people feel, how it affects people… ***To experience it gives them an insight that I don’t think a lot of people had.*** It’s like you can have as much theory as you want but when it comes to practice or practical side if you don’t understand the problem I don’t think you can advise anybody on it. WS2_ZG_Us_Magpie_003 (ZG)  something I’ve personally found quite difficult with mental health services generally. I find them quite patronising and I think having users that have also suffered or are suffering with mental health problems makes it immediately less patronising because ***you know that they know and I do believe you can’t teach that necessarily***. Not that that’s ideal for your study but like how do you teach someone the true feelings – how do you explain a feeling? That’s quite a complicated thing isn’t it so I think particularly in forums having peer support, knowing that they have also experienced it in some way or another just gets rid of some of the patronising elements of WS2_PM_Us_Starling_005 (ZG)  Yeah there’s a... specific for mental health to get advice it’s really nice people and they’re expert – them giving all experience and their struggles and how they dealt with so it’s been nice. It kind of ***made me feel less strange I suppose knowing other people are also going through it and also*** ***that they’re trying to help as well and it feels like a community kind of***. WS2_PM_Us_Starling_002 (ZG)  I get the impression that moderators in some way who are the ones who’ve experienced it from what I’ve seen on the posts ***they don’t seem to share that much personal stuff which actually I think would be a benefit to people*** who are wanting the recovery side and how they’ve done it and how they’re experiencing it so yeah I think that would be good from a moderator WS2_PM_Us_Sparrow8_035  on here there’s a lot of people going through very similar – they’ve all got their own twists on but similar mindsets or similar problems and ***it’s just very supportive to hear people – you’re not basically on your own***. WS2_PM_Us_Sparrow2_023 (ZG)  It makes you feel less isolated and alone. I think particularly mental health problems it can skew your views into making you feel like you are the only person. It’s like having an external community of other people who are suffering***, it kind of makes you feel less isolated with it and less defective*** almost because you do feel defective as a human ***but lots of people are suffering. It can’t just be you can it*** WS2_PM_Us_Starling_005 (ZG)  I can’t remember now. I think I did and I ended ***up talking to loads of people and straight away it took that stigma*** – I didn’t have a stigma about it. Well, no, that’s a lie, I did. I thought, ‘Oh my God they’re all a bunch of weirdos. There’s no way I’m talking to them,’ and it’s not the case. You come away and you feel a lot better because you think, ‘Oh God it’s not as bad as you think, ***these people are normal, they’re just like me. I’m not mad.*** WS2_PM_Us_Magpie_126 (ZG)  what you learn is that you’re not on your own, you are not alone, you are not a unique person. It doesn’t happen only to you and that’s comforting definitely. ***It gives you the feeling that it’s not me being weird or odd or whatever***. T***here are others who do suffer in a similar way so I think you are attracted to that that it’s not only you.*** I don’t think you learn much but only that part that it’s you are not unique, this has happened or is happening to others as well. WS2_PM_Us_Magpie_022 (ZG)    They’re literally just called forums on ((Name)) and I personally quite like them, especially when I can find the ones that I can relate to because some of them are made by practitioners and it’s just links to helpful websites and stuff we can use but ***I personally like the ones that other users write because I can see that there’s other people that are going through similar things***. WS2_PM_Us_Dunnock_046 (PM)  I look for having some sort of normalisation and... ***just having that reassurance of what I’m going through is normal***, that my condition – ***my mental health condition is part of me and it’s not something I should work against, if that makes sense*** WS2_PM_Us_Chaffinch_001 (ZG)  You need all these forums and things just to help your mental health ***just to make you feel a bit normal*** because you don’t fit into society when you’ve got a mental health issue. There’s people who don’t like you. When they say it’s stigmatised you’re not kidding. WS2_PM_Us_Magpie_126 (ZG)  coming back to dyslexia again because it’s a very good example. When I talk to people about dyslexia they think they’re the only people in the world that have got dyslexia. When you say, ‘Oh well I’ve got it as well.’ ‘Have you really?’ I said, ‘Yeah and these are the problems I have…’ and they’re so relieved to talk to somebody who’s got the same type of problem and understand exactly how they feel and gone through exactly what they’ve gone through because immediately ***you see them pick themselves up a bit thinking, ‘Goodness me, I didn’t realise everybody felt the same way***,’ and it’s the same with depression WS2_PM_Us_Sparrow2_083 (ZG)  I ***don’t feel alone anymore and that’s the best thing about it*** is when you go on forums and you go on these mental health support networks and you can see people, you know they’re there because they talk and they speak and it’s all words, it’s all writing you’re not alone. Sometimes you don’t always have to have that face to face person. Sometimes I think it’s better because I don’t want to – it’s a choice. I don’t want to be around people. I don’t get lonely but as soon as I get lonely I go onto the forums and I don’t feel lonely anymore. WS2_PM_Us_Magpie_126 (ZG)  It’s the general atmosphere of it which I think is the participants and ***it’s very accepting*** of anything you say like there’s nothing that would – everyone would support you on anything you said, if you see what I mean. they wouldn’t, ‘That’s your fault,’ or anything like that. I think it’s the atmosphere... ] that this is very kind, supportive... It is like counselling. I think that’s where the tone has come from where you’re giving indirect advice and listening. ***Very much listening and validating what people are saying***. WS2_PM_Us_Sparrow2_006 (ZG)  I tried to look on other places but I think the [Magpie} is probably one of the best forums basically or that group is particularly a good one because ***you’ve got the support but you’ve got that focussed lived experience***. They are people who are ***peers to you who understand you and can support***. We’re in it together and we are survivors whereas you don’t always get that with the other ones. WS2_PM_Us_Magpie_077 (ZG)  Definitely community, so ***knowing that other people on the sub cared and trying to show that care myself*** because modern society is often quite fractious. We don’t have community in the same sort of way that we used to. You don’t go to a youth club or whatever and ***so I think that’s something that uniquely peer support forums can do for people is providing that community and just that unsaid knowing of they understand*** WS2_PM_Us_Dunnock_046 (PM)  I think maybe I can say it ***made me feel less alone*** because most of the time I feel like I am alone ***so when I went to the forums I felt like I’m not alone***, I can have some engagement with some people and I felt like I’m around some people, I’m around some friends so I felt like I’m not alone. WS2_PM_Us_Jay_001 (ZG)  I thought about a particular thread it’s one of these bit odd ones like, ‘Cannot sleep,’ thread so anybody who’s awake in the middle of the night they post on this thread and they’re like, ‘Oh I can’t sleep at night,’ or something like that and then other people reply, so I guess in this illness there are a lot of people who struggle with their sleep so they can feel quite supported and not alone. Sometimes you feel like it’s dark and everybody is asleep and you’re just alone suffering by yourself but being able to access the forum and this particular thread ***it makes you feel a bit nicer I think that there are other people who are in the same position as you*** WS2_ZG_Us_Robin_009 (ZG)  I’d say because no one knows each other and there’s a reason for that which is a good thing but because of that it’s like - not a downfall but it’s both good and bad that no one knows each other because ***you can’t form any personal attachments and things because it’s not – you don’t know anyone***. For your own safety as well, you don’t know anyone so everything feels like – it’s definitely warm. I’d describe it as welcoming but there’s not much culture on there because no one knows each other and i***t’s hard to directly communicate with specific people.*** WS2_PM_Us_Dunnock_073 (PM)  a lot of the time it’s just I’m more interested in what other people say because what it can do, forums like this is I’m not on my own, I am normal or ***a terrible thing to say but gosh they’re much worse than me. I’m clearly fine***.  (same person )  You’re not the only one, there are other people out there. this is going to sound awful – comparison. ***Oh, I’m not as bad as them, it’ll be okay, I’m not the worst in the world***. … you can benchmark your experience in a way by reading other people’s and getting a feel for where you sit in relation to your own experience through the lens of other people.  I think for me it’s been a big source of purpose I would say. Like I say I’d always like to try and have some sort of service in my life so having this and the way that I guess the experience that I’d had in terms of speaking to people and hearing stories and that kind of thing on the one side it’s humbling in that you hear something different with people with different experiences ***and it’s always a reminder that you appreciate what you do have*** | **CMO – connection through understanding and acceptance**  When forums bring together people who share similar personal experiences, particularly ones stigmatized in society (context), users have access to posts that resonate for them (mechanism—resource). When they see these experiences being accepted and understood, this normalizes their mental health experiences and validates their own reactions to similar situations, and they feel like they belong in the forum (mechanism—reasoning).  If users feel safe enough to make a post (context) and receive timely, constructive, and empathetic responses from other users (mechanism—resource), they will feel personally recognized, and even more understood and accepted (mechanism—reasoning), leading to a reduction in self – stigma (outcome), and a further enhanced connection with the online community (outcome), reinforcing ongoing forum engagement (outcome). |
|  | CMO 5: when forum users post messages (context) and receive timely, constructive, and empathetic responses from other users (mechanism—resource), they will feel recognized and understood (mechanism—reasoning). This will contribute to a sense of connection (outcome) with the online community (outcome), increasing forum engagement (outcome). | It’s like you want to have a sort of friend there who is going to try and support you, you know what I mean? You don’t want it to be really official like, ‘You need to go and visit this website or ring this number,’ because people are getting pied off left, right and centre... it’s really hard to get any solid answers so rather than I think being signposted elsewhere, obviously do it when it’s needed. If say someone’s feeling suicidal then be like, ‘You need to go to A&E,’ or something like that but sitting there and actually listening and feeling WS2_PM_Us_Magpie_032 (ZG)    Sometimes they’ll be there to explain what the situation is. They’re not asking for advice, they’re not really asking anyone to listen but sometimes they just need a space for someone to say it and it’s just to say that – ***acknowledging that this has happened and your emotions are valid in what you’ve gone through*** and again it’s just [something like: 0:10:40.0] an acknowledgement that someone will say again thank you or they’ll open up again WS2_PM_Us_Chaffinch_009 (ZG)    I could be just like I haven’t got anything to offer but, ‘I’m here for you.’ Being able to listen to people when they’re feeling rubbish e***ven if it’s nothing more than just saying, ‘I hear you,’ or something that’s really personally helped me feel better in myself.*** WS2_PM_Us_Starling_005 (ZG)       \| It’s ***helped me not to be not so ashamed***. It’s helped – ***this is my story***, I’m a survivor. It helped me to be a warrior and not to be so ashamed and to hide it. It’s helped me to talk to people. It took me ages to say I was ill WS2_PM_Us_Magpie_077 (ZG) \| \| --- \| \| it’s a positive thing the fact that you can post whatever you want to ***post without feeling judged or potentially having a negative consequence.*** They don’t – people don’t tend to post negative stuff or criticisms. WS2_PM_Us_Sparrow2_002 (ZG) \|     It makes you feel less isolated and alone. I think particularly mental health problems it can skew your views into making you feel like you are the only person. It’s like having an external community of other people who are suffering, i***t kind of makes you feel less isolated with it and less defective almost because you do feel defective as a human but lots of people are suffering. It can’t just be you can it*** WS2_PM_Us_Starling_005 (ZG)    the mental health is quite isolating at times, especially if you don’t – for me I don’t talk about it outside of my house. I don’t tell people that I’ve got any mental health problems and you wouldn’t know outside of my very close family and friends, ***so it’s a way of being able to talk about it without other people judging you I suppose***. WS2_PM_Us_Sparrow2_006 (ZG)    I’ve gone through years with people I suppose masking would come to mind. I think I’ve got a problem but I can’t let them know because they won’t understand so ***whereas with ((Name)) I think they do understand because they’ve all been through it so I don’t have to mask it, I can be me.*** WS2_PM_Us_Sparrow2_003 (ZG) |  |
|  | Negative experiences | Talking about general mental health in a way and coping strategies yeah but being more open about my sexuality and identity not really because obviously ***they’ve been taken down so I don’t think I should be speaking about them*** in person at the time of me posting them. I mean as I got older I’ve been able to be more open about those conversations but when I probably should’ve been talking about it in the beginning and it being taken down quite quickly ***it kind of didn’t make you want to talk about it so it’s kind of in a way also not nice.*** *WS2_PM_Us_Dunnock_229 (ZG)*  some members are online, some members are not online which is okay but sometimes mainly when you’re going through a crisis and you want to jump on the forums and ***no one is responding even a little bit it may feel a bit hurt sometimes or maybe feel a bit dejected like, ‘Why is no one responding?’*** WS2_PM_Us_Chaffinch_001 (ZG)  I think it would very much depend on your frame of mind when you’re using the service. ***If you’re low*** and you’re looking for that support if you post something and then ***you don’t hear something back for a day or over a day*** I think that would ***compound the feeling that you have of, ‘I’m low, I’m not worth anything***. See, people aren’t responding to me. This is further evidence, WS2_PM_Us_Sparrow2_012 (ZG)  you’d see some threads that had – you’d have active users but you’d have no one responding to certain ones and it’s like when you – sometimes sharing that kind of experience can be quite difficult so if you check that after a week and there’s lots [of replies to:: 0:04:49.6] people and ***yet no one’s responded to you the feeling is you’ve gone to this place which is meant to be supportive and to listen and no one has.***  WS2_PM_Us_Chaffinch_009 (ZG)  We have talked about you know, timely - being responsive timely – yeah I think by that – that’s what I felt like they were not adhering to but other things they were good, they were good but timely responses were not that effective...Yeah they were very inconsistent and sometimes you were meant to [post: 0:41:11.2] for maybe it’s after 24 hours they have not responded so you are first to repost so [I can see: 0:41:19.4] they are ***not always available but by that they make some other people feel like they are not being attended to.*** *WS2_PM_Us_Jay_001 (ZG)* |  |
| Theory area 3: Helping others | CMO 4: users who share their personal experiences on the web (context) derive satisfaction (outcome) from the knowledge that their posts help others (mechanism—reasoning), particularly when others express gratitude (mechanism—resource). | I was kind of able to do a lot of work to aid and speed my recovery and **being able to share that with other people it just made me feel good**. It just gave me a dopamine hit I guess just from being helpful or ***thinking I was being helpful*** to others and yeah just hoping other people would feel they weren’t alone and feel that they were understood and if they found one thing helpful that I’d come across then it would be worth it WS2_PM_Us_Magpie_213 (ZG)    In a small degree it does and then f***eeling good about yourself for helping someone***. The idea of taking yourself outside of yourself so when you sit and dwell on your own problems it’s not very helpful. ***Then you go outside yourself to help someone else it makes you feel a lot better***. WS2_PM_Us_Sparrow2_006 (ZG)    The main thing I get out is just having other people who may share similar experiences and supporting each other by supporting a couple of people in particular then that ***enhances my wellbeing*** in the fact that I feel like I’m making a difference to – not a lot of people but just a few people and ***I also when I get a response back from somebody maybe give some different idea***s. If I haven’t been sleeping well try this, try that, so it’s sharing ideas and it’s just having the knowledge that if you do posts something you will get something back within sort of – well, within 24 hours normally. WS2_PM_Us_Sparrow2_002 (ZG)      Yeah so it’s done a lot since I was ill and that gives me – to ***know that other mothers are getting the help that I didn’t have it’s helping me***. ***It’s making me feel empowered because I can make changes through these forums and doing things like this now.*** WS2_PM_Us_Magpie_077 (ZG)    say a lot of them their health has improved and what they’ve done is they’ve gone on and stayed on these apps, they’ve **stayed on these forums to help other people.** That’s their main purpose because ***they’ve had probably such a bad time with their mental health and had to go through so many loopholes*** and they’ve had so many bad experiences or good and bad experiences ***they want to share that with other people*** because I know I do. I like to – if I can see somebody ask a question and nobody else has answered it I’ll try and answer them as much as I can and [pass it on WS2_PM_Us_Magpie_126 (ZG)    it sounds like they’ve been dealing with bipolar disorder for many years like 20, 30, 40 years so they’re very well – they know the illness really well and they know what can happen and stuff like that, ***so those are ones that give more than they take I think because they encourage other people*** and tell people things but I think the newer users are the ones that need more support. I guess they are asking for help from these people yeah WS2_ZG_Us_Robin_009 (ZG)    so trying to give my own sort of support or advice can feel quite ***therapeutic yourself to try and help others and especially if you’re more the recovery or remission side of it***. WS2_PM_Us_Sparrow8_035 (ZG)      It’s nice when you’re at that starting point that there are other people there that are maybe a few weeks or a few months ahead of you ***so you can see that all these people started in the same place and progression is possible***, so that’s one of the nice things about the forums. WS2_PM_Us_Dunnock_196 (ZG)      I think especially when like I say you’re using a particular forum and there’s a regular use that you can keep going back to and interacting and you see them - maybe they have an issue and y***ou see them getting past that or you see them over time become a bit more confident*** WS2_PM_Us_Chaffinch_009 (ZG)    back when I was very unwell I used to post a lot and ask a lot of advice and share my feelings and talk to people about my feelings but ***now I’m feeling better and in a more stable position I tend to use the forums so I can help others and it gives me a real sense of achievement and having some use.........( ) ....*** I feel like I make a difference for other people and it ***makes me feel less useless*** because I feel if I can help somebody who hasn’t got help like me then ***there’s still a point in me being here***. WS2_PM_Us_Starling_001 (ZG)      My mental health definitely improved when I could help other people because I think k***nowing I was helping others made me realise that those techniques were actually helping and I could try [using them as well: 0:49:07.0***] whereas sometimes I’d try them once when it wasn’t working and just scrap that idea completely but I still [give: 0:49:16.1] the idea to someone else ***in case it was helpful to them but then I think it made me realise I could come back to these things and try them over and over*** because sometimes they will work and sometimes they won’t. WS2_PM_Us_Dunnock_046 (PM)    People a lot of the times struggle with feeling like I’m not doing a good job, they’re not a good mum, not getting things right and really struggling with everything they’ve been through and I think ***knowing that just the fact that they’ve been brave enough to post something is actually really great*** so it might be – some people might not like it but to kind of say your post has been viewed this many times or sharing how many people it helped reach that the poster had. WS2_PM_Us_Magpie_213 (ZG)    I think especially when like I say you’re using a particular forum and there’s a regular use that you can keep going back to and interacting and you see them - maybe they have an issue and you see them getting past that or you see them over time become a bit more confident. I remember one particular interaction I had with someone who was really nervous, really socially anxious. They were joining university, they were really worried about how they were going to socialise and everything and it was a conversation we could have back and forth through this thread and eventually after some conversation after realising that actually no it wasn’t as scary they actually ended up going and having a fantastic time and in their head they never thought in a million years it could get to that stage because they thought that I’m too far gone, that kind of thing. WS2_PM_Us_Chaffinch_009 (ZG) | **CMO – connection through helping others**  When more experienced users (context) share their personal experiences and expertise on the web in response to requests from others (mechanism- resource) this increases their wellbeing (outcome) because it gives them a sense of purpose and value (mechanism - response) and leads them to revisit and remind themselves of how best to manage their own health (mechanism - response). When responses are met with expressions of gratitude, or recipients shows improvement in their lives (context), this sense of value is heightened, reinforcing further forum use and continued wellbeing (outcomes). However, if users read about difficult experiences that resonate for them (mechanism - resource), they may empathise deeply and want the person to receive help quickly, increasing their sense of responsibility (mechanism – response), and negatively impacting their own wellbeing (outcome). This is more likely to happen when the user is not confident in the moderators’ ability to respond and manage the situation (context). |
|  | Negative impacts of helping others | if they were posting about something quite serious like self-harm it would maybe be a bit kind of what mood they were in when they wrote it because if the person is in crisis and they type on there when they’re in crisis and it might come across quite like – well, ***I just feel really worried about them which is why I think it’s really good that they have the moderators then I know it’s not – that person will be offered support which I think is really helpful***. It is really helpful because you know it’s kind of trusting the system will help them or try and offer some kind of help but I think yeah I do get a little bit worried sometimes when I see that someone is feeling really down and needs help and stuff. I think that’s probably quite normal I guess WS2_ZG_Us_Jay_009 (ZG)    you just look at it and read it and think oh no that person up at 02:00 in the morning, they’re obviously having a nervous breakdown and they’re really upset and you just read the headline of what that person’s put. You don’t have to actually delve into reading what they’ve put, just the headline of what they’ve put so then you know not to click onto that...you don’t have to read it. ***You might just get the header and think I won’t bother reading that so that’s why I don’t bother because I think I already know them, that’s going to upset me***, so I’m not going to go and talk to that person or read what they’ve put because they seem really down and ***sometimes I do have a quick look to make sure they’ve not been ignored or to see if somebody’s spoken to them*** WS2_PM_Us_Magpie_126 (ZG)  sometimes in the back of your mind you don’t actually know the other people online either so when I was answering I would answer in a way not to identify myself still and trying not to talk to the same people regularly because I didn’t want in a way to be like – ***I don’t want to be attached to people online that I don’t know basically...I think I’d just struggle with them let’s say if they had a relapse or they were going downhill and then they didn’t reply for X amount of time on the forum, I think I’d become quite concerned and then I don’t know if that would affect me and my mental health*** and maybe go down, so that was always something else that was at the back of my mind. I didn’t want to know people too much because obviously professionals must be helping them and there’s counsellor is on (forum), they are there to deal with that. That’s not my responsibility. WS2_PM_Us_Dunnock_196 (ZG)      I think there were moments where I’d see something come through and I’d think, ***‘I’ve got to reply to that,’ and kind of dropped the things I was doing or rearranged my priorities to reply and obviously that’s not healthy*** but I think that’s a very... personal issue of mine...I was like, ‘I’m being useful,’ so it kind of fills that need for purpose that I was seeking out so yeah it was a bit of a coping mechanism in a lot of ways but yeah better than drinking I guess. WS2_PM_Us_Magpie_213 (ZG)  some of the posts that people were putting up was kind of like, ‘Hi, I’m such and such, I’d like to make some friends here,’ and it’s kind of like you think, ‘Oh but why?’ ***Hopefully they’re not spending too much time online if they’re just hoping they’re going to make friends online and then not make friends offline.*** WS2_PM_Us_Chaffinch_022 (ZG) |  |
| Theory area 4: - | No previous CMO | Sometimes you can get quite rambling posts and I’ve done similar myself where you just kind of type out your stream of thoughts without trying to curate it too much, whereas in person I think you would struggle to try and express it because you’d be trying to curate it as it’s coming out whereas typing particularly because people are good at typing or they would be doing it on their phones, ***they can just kind of ramble out their stream of conscious*** WS2_PM_Us_Starling_005 (ZG)        That’s it yeah or just to vent really sometimes. Just to put down how you’re feeling. Some people journal apparently, I don’t ***You’re sort of almost then looking in on it from other people’s point of view*** and you also get support which is helpful. Sparrow2_006    I: what impacts does writing about your own experience on the forum have on you – the actual process of writing things out?  R: It makes it solid. It makes it real and actually ***when it’s whizzing like a merry-go-round in your brain actually writing it down it kind of is there, it takes it out of your brain so much***. My doctor encouraged me to write as well. I used to just write everything down and then hand it to him and then we’d talk about it WS2_PM_Us_Magpie_077 (ZG)    If I hadn’t joined the forum I don’t think I would have been here speaking to you now... I think it’s the fact that I’ve been able to connect with people that have suffered, are suffering from the same problem as me, anxiety and depression, that I think losing people it’s – ***a lot of people when people die can’t talk to people about them*** and I found when ((Name)) first passed away that people that we both knew if they saw me out in the street they’d cross over…so they didn’t have to speak to me..., ***but I wanted to talk about ((Name)). I wanted to remember ((Name)).*** WS2_ZG_Us_Magpie_003 (ZG)    I think i***t’s actually being out of control which causes mental health*** as well because you’re not in control. Like, ‘Take these pills, they’ll do you good.’ ‘Do this therapy, it will work.’ ‘Do this…’ and they expect it and in therapy sometimes with CBT you start getting that out of control. If you don’t do your [homework: 0:32:13.7] sorry, you might get chucked off the course or your therapy will stop and things like that. You’re already scared. [They can do these things and: 0:32:23.7] it’s traumatising me so yeah I didn’t need that. ***I needed to know that I had that control*** WS2_PM_Us_Magpie_077 (ZG)    I don’t think Dunnock can access our IP address but I do know that other services can but I think that’s something that’s really unique and different about Dunnock because I think people would feel safer if our IP addresses cannot be traced because s***ometimes you feel a bit hesitant about saying for example, if you were experiencing suicidal thoughts and they wanted to express our feelings about that knowing that Dunnock may send the emergency services to our house that would make us less likely to reach out for support and talk about those feelings***. WS2_PM_Us_Dunnock_002 (ZG)      I think because you know that nobody is going to contact you off the forum and that kind of thing. I think if you’re anonymous it is easier to share things that are a bit deeper in your mind. Does that make sense? Yeah, you’re more likely to be ***more vulnerable I think if you know nobody can contact you personally***. WS2_PM_Us_Sparrow2_084 (ZG)    Yeah I think being anonymous helps me to disclose a lot of parts of myself because because I don’t normally talk to my friends – I did talk to my friends but ***I don’t disclose parts of my feelings because I’m afraid of certain repercussions***, so when I’m online and talking about things I consider very dark. I consider myself I have thoughts that I consider very dark. ***I feel more at ease knowing that it’s not going anywhere, it’s not going to damage anyone’s relationships.*** WS2_PM_Us_Chaffinch_001 (ZG)    also I think there is an option to have comments on and comments off because sometimes people don’t want to have other people’s sympathy and empathy or they don’t want people to access what they were saying and some people do want that so I think that would be a really good option for online forums... I think they don’t want other people to have their say in a way because that’s their experience or what they’re going through and they probably have a lot of, ‘Everybody’s going to be here for you, you can do this. I get what you’re doing.’ ***Sometimes people just want to share their experience and not have other people’s experiences added on top of it***. I think it’s just a preference thing for some people WS2_PM_Us_Dunnock_229 (ZG) | **CMO – Catharsis and Sense making**  When people are truly anonymous in a forum with people with shared experiences, and they can control the responses they invite from others (mechanism - resource), then they feel safe enough to post about their most challenging experiences to the forum (mechanism- response). The process of writing, and the externalization of the experiences onto the page, can bring distance and new perspectives (mechanism- response) which can fundamentally change the experience (outcome). This can happen without any response required and may rely on being able to control responses. This is particularly important for issues that are difficult to discuss face to face or with friends and family (context). |
